# Biodegradable scaffolds for enhancing vaccine delivery

**DOI:** 10.1002/btm2.10591

**Published:** 2023-08-21

**Authors:** Matthew D. Kerr, Wade T. Johnson, David A. McBride, Arun K. Chumber, Nisarg J. Shah

**Affiliations:** ^1^ Department of Nanoengineering University of California San Diego La Jolla California USA; ^2^ Chemical Engineering Program University of California San Diego La Jolla California USA

**Keywords:** compounds/materials, drug delivery, immunotherapies, medical devices, tissue engineering

## Abstract

Sustained release of vaccine components is a potential method to boost efficacy compared with traditional bolus injection. Here, we show that a biodegradable hyaluronic acid (HA)‐scaffold, termed HA cryogel, mediates sustained antigen and adjuvant release in vivo leading to a durable immune response. Delivery from subcutaneously injected HA cryogels was assessed and a formulation which enhanced the immune response while minimizing the inflammation associated with the foreign body response was identified, termed CpG‐OVA‐HAC2. Dose escalation studies with CpG‐OVA‐HAC2 demonstrated that both the antibody and T cell responses were dose‐dependent and influenced by the competency of neutrophils to perform oxidative burst. In immunodeficient post‐hematopoietic stem cell transplanted mice, immunization with CpG‐OVA‐HAC2 elicited a strong antibody response, three orders of magnitude higher than dose‐matched bolus injection. In a melanoma model, CpG‐OVA‐HAC2 induced dose‐responsive prophylactic protection, slowing the tumor growth rate and enhancing overall survival. Upon rechallenge, none of the mice developed new tumors suggesting the development of robust immunological memory and long‐lasting protection against repeat infections. CpG‐OVA‐HAC2 also enhanced survival in mice with established tumors. The results from this work support the potential for CpG‐OVA‐HAC2 to enhance vaccine delivery.

**Translational Impact Statement**

Biomaterials have been shown to be effective at mediating potent immune responses. While sustained delivery is desirable to achieve a durable immune response, the biomaterial may induce a foreign body response that could limit clinical utility. Here, we show that a biodegradable hyaluronic acid‐based scaffold vaccine mediates strong cellular and humoral immunity with a well‐tolerated foreign body response. The results support the potential of the approach  in immunotherapy.

## 1 INTRODUCTION

Biomaterial‐based vaccines have been used to address challenges associated with conventional bolus vaccination strategies. In particular, biomaterials focused on sustained release of vaccine components antigen and adjuvant have been demonstrated to induce a more potent, durable protective immune response compared with bolus vaccination.^1^, ^2^, ^3^, ^4^, ^5^, ^6^, ^7^, ^8^ These studies have strongly supported a key role of sustained release of vaccine components in enhancing the immune response. While effective at inducing an immune response, the formulation itself could include components that could result in a persistent foreign body response (FBR), such as those that use long‐lasting polymers,^2^, ^9^, ^10^ that could be limiting in clinical settings.^9^, ^11^ On the other hand, a degradable biomaterial‐based vaccine which maximizes activation of the adaptive immune responses while avoiding a long‐lasting FBR could be a potential alternative.

Hyaluronic acid (HA) is a polysaccharide‐based polymer that is abundant in tissues including skin, cartilage, and synovial fluid. It has been widely studied in biomedical applications including drug delivery as HA can be modified to form a matrix to encapsulate drugs, such as growth factors and chemotherapeutic agents, and release them in a sustained manner via controlled degradation.^12^ We have previously demonstrated that the degradation of HA can be immune‐responsive, mediated by oxidative burst of neutrophils,^12^ and is also processed by endogenous hyaluronidases^13^, ^14^ (HYAL). The degradation generates low molecular weight HA fragments which have been shown to activate toll‐like receptors (TLRs) and act as adjuvants in vaccine formulations.^4^, ^5^, ^6^

Here, we hypothesized that a HA‐based vaccine formulation would enhance immune cell activation and sustain release of encapsulated vaccine components to generate a durable protective immune response. We had previously developed a macroporous injectable HA hydrogel, termed HA cryogel, made with bioorthogonal crosslinking chemistry.^12^ HA cryogels mediated sustained release of protein therapeutics to enhance innate immune cell regeneration.^12^ In this work, we extend our previous findings to further develop HA cryogels as depots for sustained release of vaccine components. After evaluating cryogel formulations generated from commercially sourced HA by immunophenotypic and histological assessments, we selected low endotoxin HA, termed HAC2 for evaluation with encapsulated model antigen OVA, adjuvants CpG‐ODN 1826 (CpG) and granulocyte macrophage colony stimulating factor (GM‐CSF). Both CpG and GM‐CSF comparably enhanced the response to the vaccine, but GM‐CSF increased inflammation and the FBR at the injection site. We selected CpG‐loaded OVA‐HAC2 (CpG‐OVA‐HAC2) for dose ranging studies. The intensity of the adaptive immune response was dose dependent on CpG‐OVA‐HAC2 and significantly delayed in settings of neutrophil dysfunction. In post‐hematopoietic stem cell transplant (HSCT) mice, CpG‐OVA‐HAC2 enhanced antibody induction three orders of magnitude greater than dose‐matched bolus vaccination. In a B16‐OVA melanoma mouse model, prophylactic and therapeutic administration of CpG‐OVA‐HAC2 slowed the tumor growth rate and enhanced overall survival.

## 2 RESULTS

### 2.1 Synthesis and characterization of HA cryogels

**FIGURE 1:**
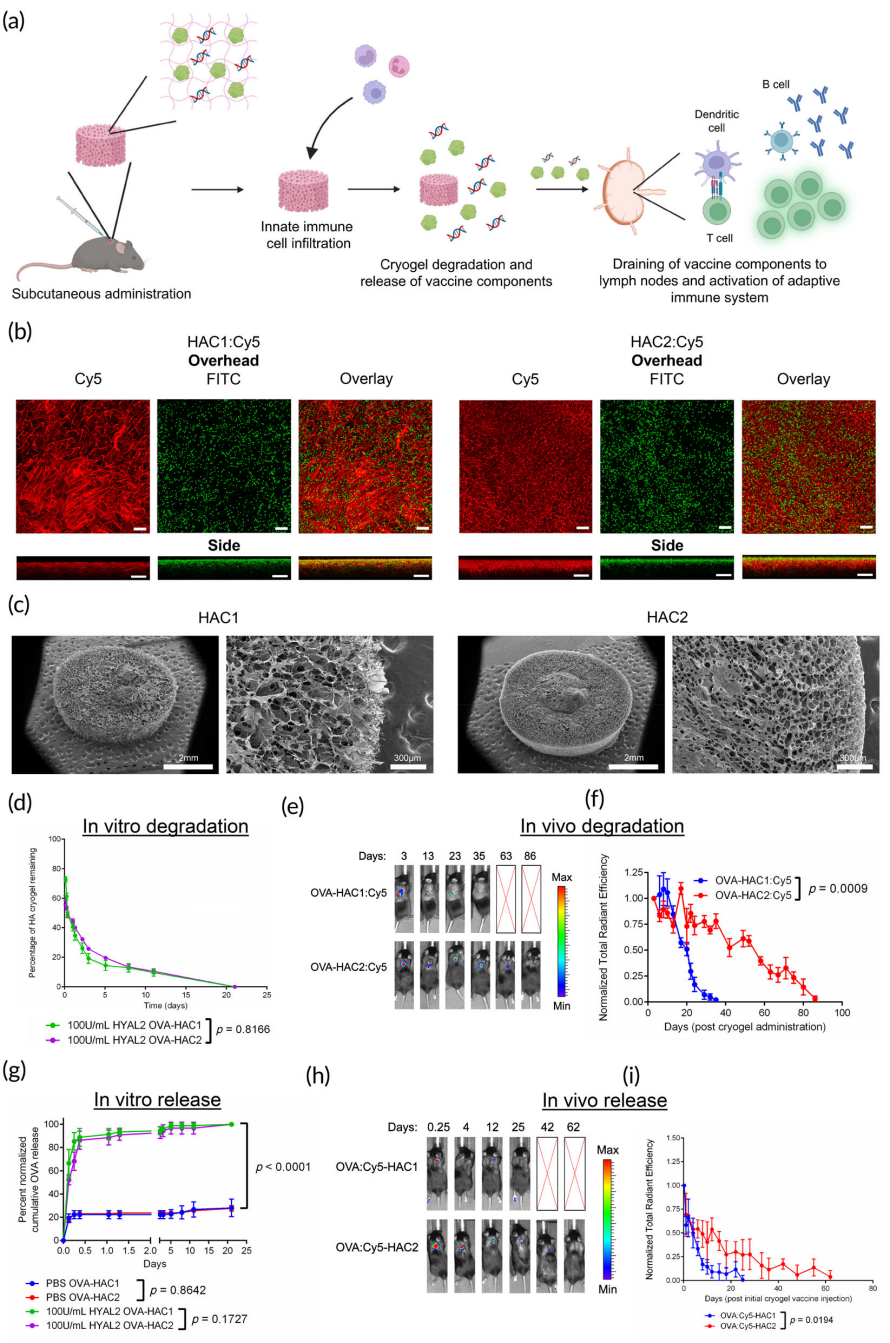
Synthesis and characterization of HA cryogels. (a) Schematic depicting HA cryogel vaccine formulation. (b) Confocal microscopy images, overhead and side views, depicting hydrated Cy5‐conjugated HAC1 (HAC1:Cy5) and HAC2:Cy5 incubated with 10 μm FITC‐labeled microparticles. Scale bar = 100 μm. (c) Scanning electron microscope (SEM) images of HA cryogels, HAC1 and HAC2. Left scale bar = 2 mm, right scale bar = 300 μm. (d) In vitro degradation kinetics of OVA‐encapsulated Cy5‐labeled HAC1 (OVA‐HAC1:Cy5) and HAC2 (OVA‐HAC1:Cy5) in hyaluronidase 2 (HYAL2) solution. (e) Representative in vivo imaging system (IVIS) fluorescence images of OVA‐HAC1:Cy5 and OVA‐HAC2:Cy5 degradation. (f) Measuring OVA‐HAC1:Cy5 and OVA‐HAC2:Cy5 degradation in vivo by quantification of total radiant efficiency normalized to initial day 3 timepoint. (g) Quantification of in vitro OVA release from OVA‐encapsulated HAC1 (OVA‐HAC1) and OVA‐HAC2 in either phosphate buffered saline or HYAL2 solution. (h) Representative IVIS fluorescence images of Cy5‐conjugated OVA (OVA:Cy5) encapsulated HAC1 (OVA:Cy5‐HAC1) and OVA:Cy5‐HAC2. (i) Measuring OVA:Cy5 release from HAC1 and HAC2 by quantification of total radiant efficiency normalized to initial 6‐h timepoint. Data in d, g represents mean ± SD of *n* = 4 cryogels. Data in f represents mean ± *SEM* of *n* = 5 mice. Data in i represents mean ± *SEM* of *n* = 4 mice. Data in (d,f,g,i) compared using two‐way ANOVA with Bonferroni multiple comparison test. In g comparison of PBS and HYAL2 release was conducted by pooling measurements for OVA‐HAC1 and OVA‐HAC2. Figure 1a created using Biorender.

HA cryogels are formulated by first conjugating either tetrazine (Tz) amine or norbornene (Nb) methylamine to HA to form tetrazine‐functionalized HA (HA‐Tz) and norbornene‐functionalized HA (HA‐Nb). Vaccine components were solubilized with the polymer solution prior to mixing and overnight incubation at −20°C to form HA cryogel vaccines (Figure 1a). HA was initially sourced from two commercial suppliers and resulting HA cryogels are referred to as HAC1 and HAC2 for HA sourced from supplier 1 and 2, respectively. For confocal imaging and in vivo degradation tracking, HA‐Nb was reacted with Tz‐Cy5 to form Cy5‐labeled HA‐Nb (Cy5‐HA‐Nb) which was mixed with HA‐Tz to form Cy5‐labeled HA cryogels, referred to as HAC1:Cy5 and HAC2:Cy5 (Figure S1a). To visualize pores and assess pore interconnectedness, HAC1:Cy5 and HAC2:Cy5 were incubated with fluorescein isothiocyanate (FITC)‐labeled 10 μm diameter melamine resin particles and imaged using confocal microscopy. FITC‐particles were co‐localized with Cy5‐labeled HA polymer to the confocal depth limit for HAC1:Cy5 and HAC2:Cy5 (Figure 1b). Next, we lyophilized HA cryogels and measured surface porosity using scanning electron microscopy (SEM; Figure 1c). Average pore diameter for HAC1 and HAC2 were found to be 59.5 ± 19.4 and 53.8 ± 15.8 μm, respectively (Figure S1b).

To test the magnitude of an adaptive immune response to the HA cryogel alone, 100 μg of OVA was encapsulated in HAC1 and HAC2 (OVA‐HAC1 and OVA‐HAC2) and in vitro degradation kinetics of OVA‐HAC1 and OVA‐HAC2 were assessed by incubating cryogels in hyaluronidase 2 (HYAL2) solution. The degradation profiles of OVA‐HAC1 and OVA‐HAC2 were comparable, with most of the degradation occurring within the first week and full degradation occurring over the course of 3 weeks (Figure 1d). To characterize in vivo degradation profile of OVA‐encapsulated HAC1:Cy5 (OVA‐HAC1:Cy5) and OVA‐encapsulated HAC2:Cy5 (OVA‐HAC2:Cy5) we utilized in vivo imaging system (IVIS). A single OVA‐HAC1:Cy5 or OVA‐HAC2:Cy5 was injected subcutaneously in the hind flank of C57Bl/6J (B6) mice. Strikingly, OVA‐HAC1:Cy5 degraded over the course of 5 weeks whereas OVA‐HAC2:Cy5 degraded over the course of 3 months (Figure 1e,f). The degradation half‐life of OVA‐HAC1:Cy5 and OVA‐HAC2:Cy5, as determined by the time to achieve a 50% reduction in fluorescence intensity was 20 ± 2 and 54 ± 5 days, respectively (Figure S1c).

We next sought to quantify the effect of degradation on OVA release. In vitro release assays for OVA‐HAC1 and OVA‐HAC2 were conducted with or without HYAL2 solution in phosphate buffered saline (PBS). In HYAL2 solution, 88.8% ± 6.6% and 86.3% ± 6.8% of OVA released from OVA‐HAC1 and OVA‐HAC2, respectively, over the first day with the remaining OVA released throughout the course of the study until the gels were fully degraded (Figure 1g). In PBS, 22.2% ± 2.8% and 23.0% ± 0.7% of OVA burst release from OVA‐HAC1 and OVA‐HAC2, respectively, within the first day with minimal release over the rest of the study (Figure 1g). The encapsulation efficiency of OVA, based on in vitro release, was 77.8% for OVA‐HAC1 and 77.0% for OVA‐HAC2. To determine in vivo OVA release kinetics, OVA was functionalized with Cy5 (OVA:Cy5) prior to encapsulation within HAC1 (OVA:Cy5‐HAC1) and HAC2 (OVA:Cy5‐HAC2) and measured using IVIS Release was quantified by measuring the attenuation of the fluorescence signal relative to the initial measurement. The release of OVA:Cy5 from HAC1 and HAC2 was sustained over multiple weeks with accelerated OVA:Cy5 release from HAC1 as compared with HAC2 (Figure 1h,i).

### 2.2 HAC1 and HAC2 are infiltrated with a distinct innate immune cell profile

**FIGURE 2:**
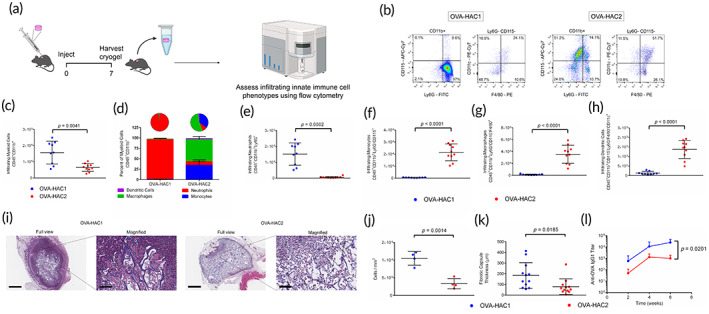
Assessment of innate immune cell response to HAC1 and HAC2. (a) Workflow schematic for assessing innate immune cell infiltration in OVA‐HAC. (b) Representative flow cytometry plots depicting gating strategy to determine cellular identity of CD45^+^CD11b^+^Ly6G^+^ (neutrophil), CD45^+^CD11b^+^Ly6G^−^CD115^+^ (monocyte), CD45^+^CD11b^+^Ly6G^−^CD115^−^F4/80^+^ (macrophage), and CD45^+^CD11b^+^Ly6G^−^CD115^−^F4/80^−^CD11c^+^ dendritic cells (DCs). (c) Quantification of total CD45^+^CD11b^+^ (myeloid) cells. (d) Infiltrating immune cells plotted as a percentage of myeloid cells. e‐h Quantification of total numbers of (e) neutrophils, (f) monocytes, (g) macrophages, and (h) DCs. (i) Hematoxylin and eosin (H&E) stained histological sections of explanted OVA‐HAC1 and OVA‐HAC2 7‐days post‐injection. Full view scale bar = 800 μm, magnified scale bar = 100 μm. (j) Quantification of cellular density in the sections from H&E slides. (k) Quantification of fibrotic capsule thickness in the sections from H&E slides. (l) Assessment of anti‐OVA IgG1 antibody titers in serum of mice which received a single injection of OVA‐HAC1 or OVA‐HAC2, administered in a prime and boost setting 11 days apart. Data in (c–h) represents mean ± SD of *n* = 9 cryogels. Data in j represents mean ± SD of *n* = 4 cryogels. Data in (k) represents mean ± SD of *n* = 12 measurements (4 measurements per cryogel). Data in (l) represents mean ± SD of *n* = 5 mice. Data in (c,e–h,j,k) compared using Student *t*‐test. Data in (l) compared using two‐way ANOVA with Bonferroni multiple comparison test. Figure 2a created using Biorender.

As endotoxin content of polymers is known to influence the immune response, the endotoxin content of HAC1 and HAC2 was quantified by measuring lipopolysaccharide (LPS) content. Endotoxin content was measured to be 7.3 × 10^−3^ EU and 6.7 × 10^−4^ EU for HAC1 and HAC2 respectively (Table 1). Innate immune cells infiltrating OVA‐HAC1 and OVA‐HAC2 were assessed 7‐days post‐injection using flow cytometry (Figures 2a,b and S2a). The viability of infiltrating cells (Aqua Zombie negative) was consistently greater than 95% in OVA‐HAC1 and OVA‐HAC2 (Figure S2b). Total CD45^+^CD11b^+^ (myeloid) cells were 2.4‐fold higher in OVA‐HAC1 compared with OVA‐HAC2 (Figure 2c). Notably, CD45^+^CD11b^+^Ly6G^+^ (neutrophil) cells constituted most of the cellular infiltrates in OVA‐HAC1 but were nearly absent in OVA‐HAC2 (Figure 2d,e). Conversely, OVA‐HAC1 had minimal CD45^+^CD11b^+^Ly6G^−^CD115^+^ (monocyte) and CD45^+^CD11b^+^Ly6G^−^CD115^−^F4/80^+^ (macrophage) cells whereas monocytes and macrophages constituted a majority of cellular infiltrates in OVA‐HAC2 (Figure 2d,f,g). CD45^+^CD11b^+^Ly6G^−^CD115^−^F4/80^−^CD11c^+^ (dendritic) cells (DCs) were sparsely found in both OVA‐HAC1 and OVA‐HAC2 (<3% of total myeloid cells), but on average higher in OVA‐HAC2 (Figure 2d,h).

**TABLE 1:** Endotoxin content of HA suppliers.

HA supplier 1			
Material	Sample 1 (EU/mL)	Sample 2 (EU/mL)	Sample 3 (EU/mL)
HA‐Tz	0.287	0.325	0.318
Cy5‐HA‐Nb	0.172	0.175	0.174
HA cryogel average endotoxin content	7.3 × 10^−3^ EU		

HA supplier 2			
Material	Sample 1 (EU/mL)	Sample 2 (EU/mL)	Sample 3 (EU/mL)
HA‐Tz	0.013	0.015	0.020
Cy5‐HA‐Nb	0.034	0.0270	0.026
HA cryogel average endotoxin content	6.7 × 10^−4^ EU		

*Note*: Measured endotoxin content of HA‐Tz and Cy5‐HA‐Nb from HA supplier 1 and HA supplier 2. The expected HA cryogel average endotoxin content was calculated from the measurements.

Cryogels were explanted from mice 7‐days post‐injection for histomorphometric analysis using hematoxylin and eosin (H&E). Both OVA‐HAC1 and OVA‐HAC2 were infiltrated and encased in a fibrotic capsule (Figure 2i). Cellularity and capsule thickness were increased in OVA‐HAC1 (Figure 2j,k). In a separate cohort of mice, we measured the host immune response to OVA‐HAC1 and OVA‐HAC2. Mice received a single subcutaneous injection of OVA‐HAC1 or OVA‐HAC2 each in a prime and boost setting 11‐days apart and were bled at pre‐determined timepoints post‐prime. OVA‐HAC1 induced higher anti‐OVA IgG1 antibody titers compared with OVA‐HAC2 (Figure 2l).

Next, we sought to determine whether the aforementioned differences in innate immune cell infiltration and anti‐OVA antibody titers might be due to differences in endotoxin content. We added LPS, the major constituent of endotoxin, in HA‐Tz and Cy5‐HA‐Nb from to generate OVA‐HAC2 with endotoxin content of 5.2 × 10^−3^ EU (low‐LPS) and 5.2 × 10^−2^ EU (high‐LPS), corresponding to ~80% and 800% of OVA‐HAC1 endotoxin content (Table S1). Innate immune cell infiltration into OVA‐HAC2 with LPS was assessed 7‐days post‐injection and compared with OVA‐HAC2 without added LPS. Inclusion of LPS had no effect on total myeloid, neutrophil, monocyte, macrophage, or DC infiltration in OVA‐HAC2 (Figure S2c–h). Mice were administered OVA‐HAC2, low‐LPS OVA‐HAC2, and high‐LPS OVA‐HAC2 in a prime and boost setting 11‐days apart and were bled at pre‐determined timepoints. No significant differences were quantified with anti‐OVA IgG1 antibody titers between any of the test groups (Figure S2i). Based on these results, we selected HAC2 for further assessment.

### 2.3 Encapsulation of adjuvants enhances HA cryogel‐based vaccine efficacy and alters foreign body response

To assess the effect of including an adjuvant in OVA‐HAC2, we selected CpG‐ODN 1826 (CpG, 100 μg), a TLR9 agonist,^15^, ^16^, ^17^ and granulocyte‐colony stimulating factor (GM‐CSF, 1 μg), a DC maturation factor^18^, ^19^, ^20^, ^21^ and alternative adjuvant.^22^, ^23^, ^24^ GM‐CSF and CpG were encapsulated in OVA‐HAC2 (GMCSF‐CpG‐OVA‐HAC2) and in vitro release studies were conducted with or without HYAL2 solution in PBS. In HYAL2 solution, 88.7% ± 4.5% and 97.1% ± 0.4% of CpG and GM‐CSF, respectively, released from OVA‐HAC2 over the first day with the remaining release throughout the course of the study with cryogel degradation (Figure S3a,b). In PBS, 53.8% ± 7.1% and 15.8% ± 3.6% of CpG and GM‐CSF, respectively, burst release from the cryogels over the course of the first day with minimal release over the rest of the study (Figure S3a,b). From the release in PBS, encapsulation efficiency was calculated to be 46.2% and 84.2% for CpG and GM‐CSF, respectively.

**FIGURE 3:**
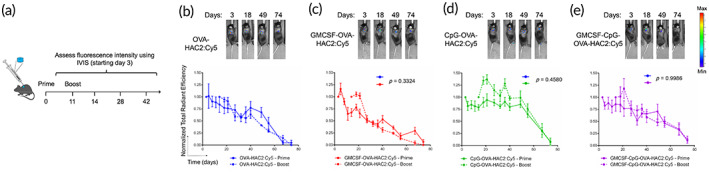
HA cryogel degradation is independent of encapsulated adjuvants. (a) Overview schematic depicting for in vivo degradation study. (b–e) Representative IVIS fluorescence images of cryogel degradation and quantification by measuring total radiant efficiency normalized to initial day 3 timepoint of (b) OVA‐HAC2, (c) GMCSF‐OVA‐HAC2, (d) CpG‐OVA‐HAC2, and (e) GM‐CSF and CpG encapsulated OVA‐HAC2 (GMCSF‐CpG‐OVA‐HAC2). Data in (e–h) represents mean ± *SEM* of *n* = 5 mice. Data in e‐h compared two‐way ANOVA with Bonferroni multiple comparison test on prime vaccine degradation curves. Figure 3a created using Biorender.

Three OVA‐HAC2 adjuvanted formulations were made by inclusion of either CpG (CpG‐OVA‐HAC2), GM‐CSF (GMCSF‐OVA‐HAC2), or both (GMCSF‐CpG‐OVA‐HAC2) and degradation kinetics were assessed and compared with OVA‐HAC2:Cy5 (Figure 3a). All components were tested for endotoxin content (Table S2). Mice were administered a prime and boost with adjuvanted OVA‐HAC2:Cy5 formulations or OVA‐HAC2:Cy5 11‐days apart. Degradation profile was comparable between all groups and cryogels were fully degraded 10‐weeks post‐injection (Figure 3b–e).

**FIGURE 4:**
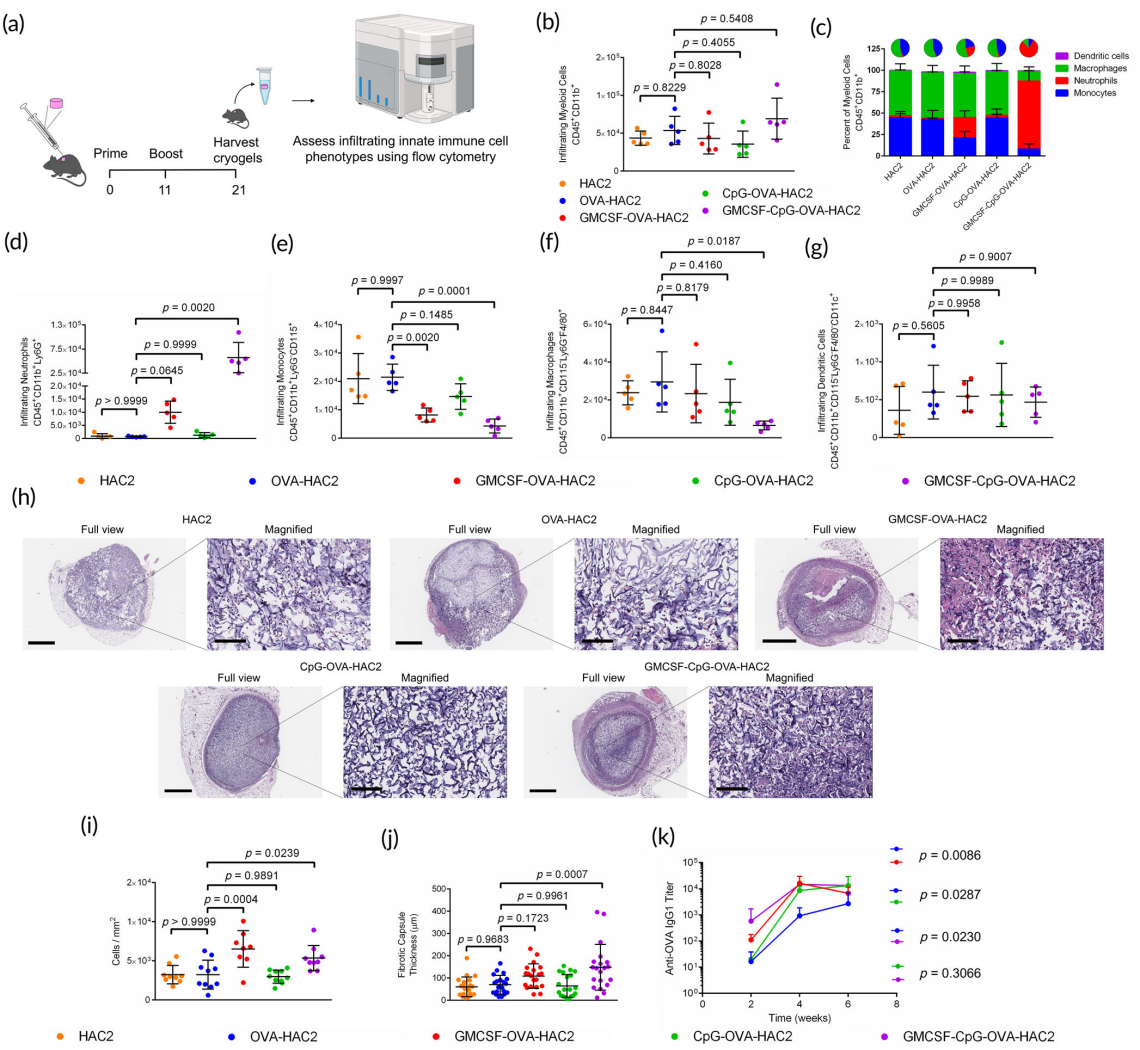
Encapsulation of adjuvants alters foreign body response. (a) Workflow schematic for assessing innate immune cell infiltration in HAC2, OVA‐HAC2, GM‐CSF and OVA encapsulated HAC2 (GMCSF‐OVA‐HAC2), CpG and OVA encapsulated HAC2 (CpG‐OVA‐HAC2), and GM‐CSF, CpG, and OVA encapsulated HAC2 (GMCSF‐CpG‐OVA‐HAC2). (b) Quantification of total CD45^+^CD11b^+^ (myeloid) cells in cryogels removed 10‐days post‐injection. (c) Infiltrating immune cell lineages plotted as a percentage of myeloid cells in cryogels removed 10‐days post‐injection. d‐g Quantification of total numbers of (d) CD45^+^CD11b^+^Ly6G^+^ (neutrophils), (e) CD45^+^CD11b^+^Ly6G^−^CD115^+^ (monocytes), (f) CD45^+^CD11b^+^Ly6G^−^CD115^−^F4/80^+^ (macrophages), and (g) CD45^+^CD11b^+^Ly6G^−^CD115^−^F4/80^−^CD11c^+^ (dendritic) cells (DCs) in cryogels removed 10‐days post‐injection. (h) Hematoxylin and eosin (H&E) stained histological sections of explanted OVA‐HAC2, GMCSF‐OVA‐HAC2, CpG‐OVA‐HAC2, and GMCSF‐CpG‐OVA‐HAC2 10‐days post‐injection. Full view scale bar = 800 μm, magnified scale bar = 100 μm. (i) Quantification of cellular density in the sections from H&E slides. (j) Quantification of fibrotic capsule thickness in the sections from H&E slides. (k) Assessment of anti‐OVA IgG1 antibody titers in serum of mice which received OVA‐HAC2, GMCSF‐OVA‐HAC2, CpG‐OVA‐HAC2, or GMCSF‐CpG‐OVA‐HAC2 administered in a prime and boost setting 11‐days apart. Data in (b–g) represents mean ± SD of *n* = 5 cryogels. Data in (i) represents mean ± SD of *n* = 8–10 cryogels. Data in (j) represents mean ± SD of *n* = 20 measurements (4 measurements per cryogel). Data in (k) represents mean ± SD of *n* = 5 mice. Data in (b,e–g,i,j) compared using one‐way ANOVA with Dunnett multiple comparison. Data in (d) was compared using Kruskal–Wallis test with Dunnett multiple comparison. Data in (k) was compared using two‐way ANOVA with Bonferroni multiple comparison test. Figure 4a created using Biorender.

Innate immune cell infiltrates in the HAC2 and OVA‐HAC2 vaccine formulations were compared 10‐ and 21‐days after injection (Figure 4a). The viability of infiltrating cells was consistently >93% in all groups 10‐days after injection (Figure S4a). In cryogels removed 10‐days after injection, total myeloid cell infiltration was similar between HAC2, OVA‐HAC2, and all adjuvanted OVA‐HAC2 formulations (Figure 4b). The fraction of Ly6G^+^ neutrophils was enhanced in both GMCSF‐OVA‐HAC2 and GMCSF‐CpG‐OVA‐HAC2 and constituted the majority of infiltrating cells in GMCSF‐CpG‐OVA‐HAC2 (Figure 4c). Neutrophil cell counts were 14.5‐ and 83.2‐fold higher in GMCSF‐OVA‐HAC2 and GMCSF‐CpG‐OVA‐HAC2, respectively, compared with OVA‐HAC2 (Figure 4d). On the other hand, neutrophil count in CpG‐OVA‐HAC2 and OVA‐HAC2 was similar (Figure 4d). Monocytes were lower in all adjuvanted OVA‐HAC2 formulations compared with OVA‐HAC2. However, GMCSF‐CpG‐OVA‐HAC2 had the lowest number of monocytes (Figure 4e). Macrophage infiltration was modestly lower in GMCSF‐CpG‐OVA‐HAC2, but similar in the other formulations (Figure 4f). DC infiltration was similar between the test groups and constituted <3% of total cellular infiltrates in all formulations (Figure 4c,g). In the cryogel formulations removed 21‐days after injection, live cell percentages were >75% on average in all groups. Myeloid cell infiltration and the constituent cells of the infiltrates were similar between groups (Figure S4b–h).

HAC2, OVA‐HAC2, and adjuvanted OVA‐HAC2 formulations were administered in mice and removed 10‐days post injection for histomorphometry‐based H&E staining. All formulations were infiltrated with cells and encased in a fibrotic capsule (Figure 4h). The cellularity was greater the OVA‐HAC2 vaccine formulations which included GM‐CSF as a constituent (Figure 4i). However, only mice which received GMCSF‐CpG‐OVA‐HAC2 had increased capsule thickness (Figure 4j).

Initial immunization studies were conducted without HA in which mice received a single prime and boost bolus subcutaneous injection of vaccine components 11‐days apart. Mice received either (1) OVA only, (2) OVA + GM‐CSF, (3) OVA + CpG, or (4) OVA + GM‐CSF + CpG and periodically bled post‐prime to assess anti‐OVA IgG1 antibody concentrations (Figure S5a). OVA + GM‐CSF and OVA + GM‐CSF + CpG enhanced antibody titers compared with OVA only (Figure S5b). OVA + CpG generated a minimal antibody response, comparable to mice which received OVA only (Figure S5b).

Next, the aforementioned OVA‐HAC2 vaccine formulations were administered to mice in a prime and boost 11‐days apart. Assessments of anti‐OVA IgG1 antibody titers showed a significant difference between OVA‐HAC2 and all other adjuvanted formulations (Figure 4k). There was no difference in antibody titers between any adjuvanted OVA‐HAC2 formulations (Figure 4k). The same cohorts of mice were used to measure degradation of the OVA‐HAC2:Cy5 formulations using IVIS. In a separate study, mice were administered prime and boost adjuvanted OVA‐HAC2 and OVA‐HAC2 only and sacrificed 3‐weeks post prime to assess CD45^+^B220^−^CD8^+^SIINFEKL^+^ cells (OVA‐specific cytotoxic T cell) in both the draining axillary lymph nodes (LNs) and spleen (Figure S5c,d). The test groups and unvaccinated mice had comparable antigen‐specific cytotoxic T cells in the lymph node and spleens (Figure S5e,f).

We concluded that all adjuvanted OVA‐HAC2 vaccine formulations comparably enhanced antigen‐specific adaptive immune response. However, formulations that included GM‐CSF were associated with an exacerbated the foreign body response. Therefore, we selected CpG‐OVA‐HAC2 for further studies.

### 2.4 CpG‐OVA‐HAC2 induces a dose dependent adaptive immune response

**FIGURE 5:**
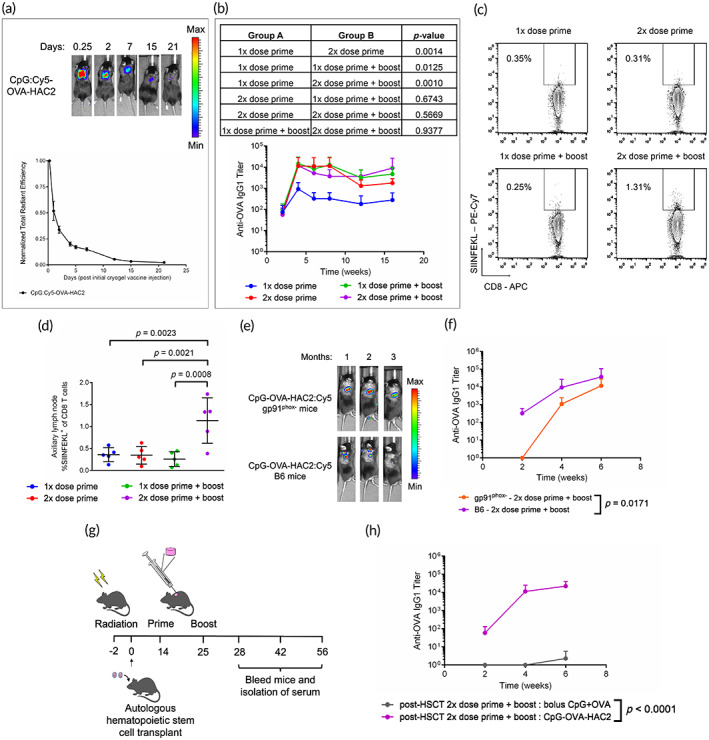
Adaptive immune response to HA cryogel vaccine is dose‐responsive. (a) Representative in vivo imaging system (IVIS) fluorescence images of Cy5‐labeled CpG (CpG:Cy5) and OVA encapsulated within HA cryogels from supplier 2 (CpG:Cy5‐OVA‐HAC2) and measuring degradation by quantification of total radiant efficiency normalized to initial 6‐h timepoint. (b) Assessment of anti‐OVA IgG1 antibody titers in serum of mice which received CpG‐OVA‐HAC2 administered as a single dose prime, two‐dose prime, single dose prime and boost administered 11‐days apart, or two‐dose prime and boost administered 11‐days apart. (c) Representative flow cytometry plots in axillary draining lymph nodes (LNs) to depicting gating strategy to assess CD45^+^B220^−^CD8^+^SIINFEKL^+^ cells (OVA‐specific cytotoxic T cells). (d) Percentage of OVA‐specific cytotoxic T cells of total CD45^+^B220^−^CD8^+^ cells (cytotoxic T cells) in axillary draining LNs. (e) Representative IVIS fluorescence images of CpG and OVA encapsulated within Cy5‐conjugated HAC2 (CpG‐OVA‐HAC2:Cy5) in B6 and gp91^phox−^ mice. (f) Assessment of anti‐OVA IgG1 antibody titers in serum of B6 and gp91^phox−^ mice which received two CpG‐OVA‐HAC2:Cy5 as a prime and boost. (g) Overview schematic for assessing anti‐OVA IgG1 antibody titers post autologous hematopoietic stem cell transplant (HSCT) mice (h) Anti‐OVA IgG1 antibody titers in serum of mice following two prime and boost vaccination of either two bolus CpG + OVA vaccination or two CpG‐OVA‐HAC2 post‐HSCT. Data in (a) represents mean ± *SEM* of *n* = 5 mice. Data in (b,d,f) represents mean ± SD of *n* = 5 mice. Data in (h) represents mean ± SD of *n* = 7 mice. Data in (b,f,h) were compared pairwise using two‐way ANOVA with Bonferroni multiple comparison test. Data in (d) was compared using one‐way ANOVA with Dunnett multiple comparison. Figure 5g created using Biorender.

We evaluated the release of CpG from CpG‐OVA‐HAC2 in vivo. Cy5‐labeled CpG (Cy5:CpG) was encapsulated within OVA‐HAC2 to from CpG:Cy5‐OVA‐HAC2. Release was assessed using IVIS. CpG:Cy5 release was sustained over a period of 3 weeks with 85.0% ± 3.1% of release occurring within the first week (Figure 5a). The effect of vaccine dose escalation on the adaptive immune response was assessed by increasing the number of CpG‐OVA‐HAC2 administered. Mice received either a single dose prime, two‐dose prime, single dose prime and boost, or two‐dose prime and boost. Assessment of anti‐OVA IgG1 antibody titers showed that a single prime vaccination induced significantly lower antibody titers than mice which received higher doses (Figure 5b). Notably, there was no difference in anti‐OVA IgG1 antibody titers in mice which received any of the other higher vaccine doses (Figure 5b). CD45^+^B220^−^CD8^+^SIINFEKL^+^ cells (OVA‐specific cytotoxic T cells) were assessed 3‐weeks post‐prime in the draining axillary lymph nodes (LNs) (Figure 5c). Only mice which received two CpG‐OVA‐HAC2 as both a prime and boost had a significantly elevated percentage of OVA‐specific cytotoxic T cells in axillary LN compared with other groups (Figure 5d). There was no difference in OVA‐specific cytotoxic T cell percentage in any of the groups in the spleen 3‐weeks post prime (Figure S6).

To better study the effect of polymer degradation and the development of an adaptive immune response, we utilized gp91^phox−^ mice which have neutrophils that lack the ability to perform oxidative burst.^12^, ^25^, ^26^ We have also previously demonstrated that HA cryogel degradation in this model is impaired.^12^ Two CpG‐OVA‐HAC2:Cy5 were administered in gp91^phox−^ or B6 mice as a prime and boost 11‐days apart. IVIS measurements on mice showed minimal degradation of CpG‐OVA‐HAC2:Cy5 in gp91^phox−^ mice and complete degradation in B6 mice (Figure 5e). The same cohorts of mice were bled at predetermined timepoints for assessment of anti‐OVA IgG1 antibody titers. There was a significant delay in the development of anti‐OVA IgG1 antibodies in gp91^phox−^ mice with none having detectable anti‐OVA IgG1 antibody titers two‐weeks post prime (Figure 5f).

To demonstrate the utility of CpG‐OVA‐HAC2 in mediating enhanced immunity, we measured the effect of treating immunodeficient mice post autologous hematopoietic stem cell transplant (HSCT) in B6 mice. Mice received lethal radiation followed by HSCT consisting of both 15 M whole bone marrow cells and 10 M splenocytes 2‐days later (Figure 5g). Two CpG‐OVA‐HAC2:Cy5 or bolus CpG + OVA were administered to mice in a prime and boost 11‐days apart and bled at predetermined timepoints (Figure 5g). Mice that received CpG‐OVA‐HAC2 had measurable anti‐OVA IgG1 antibody titers whereas there were no detectable anti‐OVA IgG1 antibodies in mice which received bolus vaccination (Figure 5h). The immunization study was repeated in the context of post‐allogenic HSCT, with BALB/cJ donor mice and B6 recipients. All the mice in this study succumbed to graft‐versus‐host disease‐like pathology within 5‐weeks post‐HSCT. Blood was collected once at 2‐weeks post prime, 4‐weeks post‐HSCT. There were no detectable anti‐OVA IgG1 antibodies in either group.

### 2.5 CpG‐OVA‐HAC2 mediates prophylactic protection in a B16‐OVA melanoma mouse model

**FIGURE 6:**
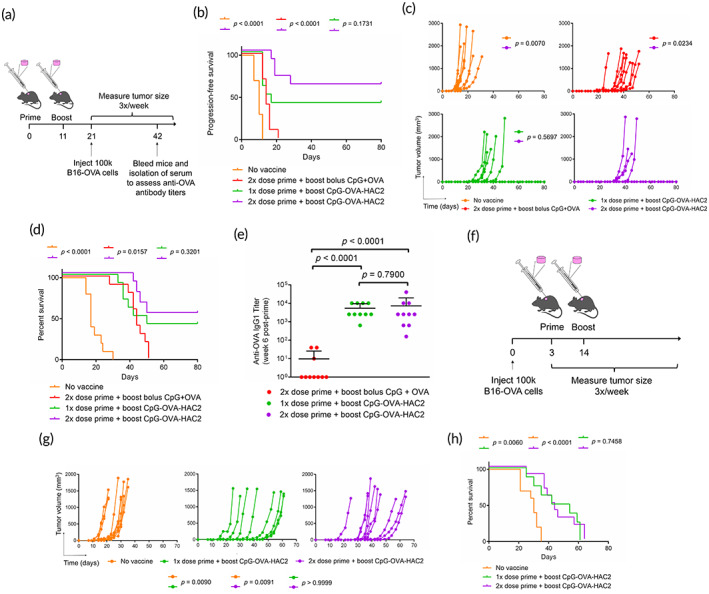
CpG‐OVA‐HAC2 provides protection against B16‐OVA melanoma. (a) Overview schematic for assessing prophylactic immunization in mediating protection against B16‐OVA melanoma. (b) Progression‐free survival, (c) tumor volume measured in individual mice, and (d) overall survival. Mice were inoculated with 100K B16‐OVA melanoma cells administered subcutaneously either in unvaccinated mice, or after two‐dose bolus, single dose CpG‐OVA‐HAC2, and two‐dose CpG‐OVA‐HAC2 administered as a prime and boost. (e) Quantification of anti‐OVA IgG1 antibody titers in serum of vaccinated mice 6‐weeks post prime and 3‐weeks post tumor inoculation. (f) Overview schematic for assessing therapeutic immunization in mediating protection against B16‐OVA melanoma. (g) Tumor volume measured in individual mice and (h) overall survival. Data in (b,c,d) represents *n* = 10 mice. Data in (e) represents mean ± SD of *n* = 10 mice. Data in (g,h) represents *n* = 8–10 mice. Data in (b,d,h) were compared pairwise using log‐rank test. Data in (c,g) was compared using Kruskal–Wallis test with Dunnett multiple comparison of area under the curve. Data in (e) was compared using one‐way ANOVA with Dunnett multiple comparison. Figure 6a,f created using Biorender.

We sought to assess whether CpG‐OVA‐HAC2 enhanced prophylactic protection in a mouse melanoma model. Mice were administered either one or two CpG‐OVA‐HAC2 or two bolus CpG + OVA injections in a prime and boost setting. Mice received 100,000 B16‐OVA cells administered subcutaneously 3‐weeks post prime or in unvaccinated mice and tumor growth rate and survival were compared (Figure 6a). In all unvaccinated mice, tumors were visible 2‐weeks post tumor inoculation with rapid tumor growth thereafter (Figure 6b,c). In mice that received two bolus CpG + OVA injections, tumor growth was delayed and were visible in all mice 3‐weeks post tumor inoculation (Figure 6b). In contrast, only 60% of the mice that received a single CpG‐OVA‐HAC2 as prime and boost had visible tumors. A total of 40% of the mice that received two CpG‐OVA‐HAC2 as prime and boost each had visible tumors 4‐weeks post tumor inoculation (Figure 6b). In all vaccinated mice, tumor growth rate was significantly reduced and increased the mean survival time (Figure 6c). Overall survival was dose responsive to CpG‐OVA‐HAC2. Mice that received two CpG‐OVA‐HAC2 as a prime and boost had the highest overall survival of 60% (Figure 6d). Mice that received a single CpG‐OVA‐HAC2 had an overall survival of 40% whereas mice receiving bolus vaccination and unvaccinated mice all succumb to B16‐OVA melanoma (Figure 6d). Vaccinated mice were bled 6‐weeks post prime, 3‐weeks post tumor inoculation, and mice which received bolus CpG‐OVA‐HAC2 had lower anti‐OVA IgG1 antibody titers than mice which received CpG‐OVA‐HAC2 (Figure 6e). Mice which survived B16‐OVA melanoma challenge were re‐challenged with 100K B16‐OVA cells three‐months post initial inoculation. All mice survived rechallenge without visible tumors for at least 40 days post re‐challenge. Next, we assessed whether CpG‐OVA‐HAC2 would provide therapeutic protection against murine melanoma. Mice were administered 100,000 B16‐OVA cells administered subcutaneously 3‐days prior to one or two CpG‐OVA‐HAC2 in a prime and boost setting (Figure 6f). CpG‐OVA‐HAC2 administered therapeutically slowed tumor growth and prolonged survival as compared with unvaccinated mice (Figure 6g,h). Mice that received a single CpG‐OVA‐HAC2 as a prime and boost survived 45.9 ± 13.8 days and mice that received two CpG‐OVA‐HAC2 as a prime and boost survived 45.5 ± 12.0 days post‐tumor challenge. In contrast, unvaccinated mice only survived 28.5 ± 5.3 days post‐tumor challenge (Figure 6h).

## 3 DISCUSSION

Here, we demonstrate that a hyaluronic acid cryogel‐based biodegradable vaccine mediates sustained release of vaccine components, enhances antigen‐specific adaptive immunity in healthy and immunodeficient mice, and provides protection against B16‐OVA melanoma in a vaccine dose‐dependent manner. The source of material and selection of adjuvants had a significant impact on the infiltrating innate immune cell subsets that constitute the FBR. We demonstrate that CpG‐OVA‐HAC2 formulation enhances anti‐OVA adaptive immune response without significantly altering the FBR and that the adaptive immune response was enhanced with escalating vaccine doses. The effect of high‐dose Cpg‐OVA‐HAC2 was studied in immune deficient models. In gp91^phox−^ mice, the degradation of CpG‐OVA‐HAC2 was delayed which resulted in slowed activation of the adaptive immunity. In post‐HSCT mice, adaptive immune activation with CpG‐OVA‐HAC2 was greatly enhanced as compared with bolus CpG + OVA vaccination, highlighting the enhancement in adaptive immune response even in immunodeficient contexts. Lastly, enhancement in immune response was assessed in both prophylactic and therapeutic vaccine studies involving B16‐OVA melanoma, in which the CpG‐OVA‐HAC2 slowed tumor growth and enhanced survival relative to unvaccinated mice and mice receiving bolus vaccination.

HA was selected as the polymer as it is ubiquitous in the ECM and has a long history of use as a biodegradable material to effectively facilitate sustained drug delivery. As an ECM component, HA provides cues to regulate inflammation and tissue repair.^27^ In the context of tissue damage or injury, HA undergoes degradation resulting in the production of low molecular weight fragments.^28^, ^29^ These fragments are known to activate innate immune cells, particularly monocytes, macrophages, and DCs, through the activation of TLR2 and TLR4.^4^, ^29^, ^30^, ^31^, ^32^ The downstream signaling of TLR activation results in secretion of TNF‐α, IL‐12, and IL‐1β which promote inflammation.^33^, ^34^ Additionally, HA fragments have been shown to induce expression of matrix metalloproteinases, which degrade ECM components and facilitate tissue remodeling.^35^, ^36^ Thus, the use of HA in biomaterials‐based vaccine not only provides sustained release of vaccine components but also potentially offers additional cues to regulate inflammation. The use of HA is further supported by other pre‐clinical vaccine studies which have utilized HA as an adjuvant in their vaccine formulations.^4^, ^5^, ^6^ Consistent with prior work, our results showing that HA cryogel encapsulating OVA alone elicits anti‐OVA IgG1 antibody titers without an additional adjuvant.^4^

Seeking to assess the effect of HA from different sources, we tested the effect of formulating HA cryogels from two commercial vendors. HA cryogels from both vendors had comparable pore size and morphology, in vitro degradation kinetics, and in vitro OVA release kinetics. There were differences in endotoxin levels, as determined by measuring LPS content, with HAC1 having 10.8‐fold higher endotoxin levels than HAC2. However, the endotoxin levels of HAC1 and HAC2 were well below FDA guidelines for implantable devices.^37^, ^38^ When cryogels were administered, OVA‐HAC1 induced higher anti‐OVA IgG1 titers than OVA‐HAC2 but was associated with a neutrophil dominated FBR with enhanced capsule thickness. However, when we added external LPS into HAC2 such that the resulting endotoxin levels were ~80% and 800% of those in HAC1, there were no significant differences in anti‐OVA IgG1 antibody titers and FBR when compared with OVA‐HAC2 without added LPS. These results suggest that increased LPS content alone in OVA‐HAC1 cannot explain the differences in anti‐OVA IgG1 antibody titers and FBR. These results suggest the potential of other components that constitute endotoxin which can also act as TLR agonists.^39^, ^40^, ^41^

Inspired by previously reported biomaterial‐based vaccine formulations, we tested the effect of encapsulating the adjuvants CpG and GM‐CSF.^1^, ^2^, ^9^, ^42^ CpG is a clinically approved TLR9 agonist and has also been extensively studied for use in other vaccine formulations.^43^, ^44^ GM‐CSF is a clinically approved agent for bone marrow recovery and has also been studied as a potential adjuvant in vaccine formulations.^45^ In contrast to other common TLR agonists such as Monophosphoryl‐Lipid A (MPLA) or squalene, CpG and GM‐CSF are water soluble and amenable for encapsulation in HA cryogels. Avoiding the need for organic solvents, solubilizers and stabilizers reduces interference with the crosslinking process. If needed, encapsulating multiple TLR agonists, for example CpG and poly(I:C), may represent a strategy for improving immunogenicity of the HAC2‐based vaccine formulation.^46^, ^47^

In our studies, inclusion of either GM‐CSF or CpG into OVA‐HAC2 enhanced anti‐OVA IgG1 antibody titers comparably and inclusion of both did not further enhance anti‐OVA IgG1 antibody titers. When we assessed innate immune cell infiltration in the various vaccine formulations, inclusion of GM‐CSF lead to enhanced neutrophil infiltration, consistent with other reports. Supporting these results, analysis of H&E stained HAC2 vaccine formulations showed an increase in cellularity with formulations including GM‐CSF. As the inclusion of GM‐CSF in the vaccine increased inflammation at the injection site and did not further improve the immune response, CpG‐OVA‐HAC2 was chosen for further evaluation.

Our previous finding of the importance of degradation in mediating release of encapsulated components^12^ was further validated in the present work. In vitro release in PBS and HYAL2 solutions showed an initial burst release followed by a period of sustained release. Burst release in PBS is expected and is observed in nearly all cryogel‐based drug delivery systems. The burst release in HYAL2 solution can be attributed to rapid initial degradation of the HA cryogels, during which ~50% of gel degraded after first 9‐h for HAC1:Cy5 and HAC2:Cy5, respectively. In vivo, the slower rate of degradation of HA cryogels corresponded to more sustained release of encapsulated vaccine components.

CpG‐OVA‐HAC2 was administered in immunodeficient mice to assess the mechanism and potential enhancement of vaccine efficacy. Consistent with our previous work, the degradation of HA cryogels in gp91^phox−^ mice was impaired.^12^ Moreover, the induction of anti‐OVA IgG1 antibody titers in the gp91^phox−^ were significantly delayed, supporting the need for release of vaccine components. Post‐HSCT Mice which received CpG‐OVA‐HAC2 injections developed robust anti‐OVA IgG1 antibody titers starting 2‐weeks post‐prime whereas mice which received bolus vaccination only developed failed to develop a detectable adaptive immune response until 6‐weeks post‐prime. These results support the utility of CpG‐OVA‐HAC2 in generating a robust adaptive immune response in immunodeficient mice.

Although two dose prime and boost was more effective at inducing a greater cellular and humoral immune response than a single dose prime and boost, the enhancement did not significantly change the efficacy of prophylactic prevention and therapeutic treatment of tumors between the two groups, as measured by overall survival. This observation might reflect a limitation in the effectiveness of CpG‐OVA‐HAC2 alone as a monotherapy and is consistent with past reports of other cancer vaccines.^7^, ^48^, ^49^, ^50^ To potentially improve cancer vaccine efficacy, particularly in established tumors, combining standard‐of‐care therapy, for example, chemotherapy or checkpoint inhibitors, with cancer vaccines has proven to be an effective strategy to prolong survival compared with either therapy alone.^2^, ^51^, ^52^ The distinct mechanisms by which these agents operate might leverage both pre‐existing tumor‐specific immune cells and generate new anti‐tumor immunity for a potentially stronger and more durable response.

The results from this study show that by sustaining release of vaccine components, CpG‐OVA‐HAC2 induces a more potent adaptive immune response compared with conventional bolus vaccination. These results are consistent with those from other biodegradable injectable scaffold systems. This includes an in situ crosslinked polymer‐nanoparticle hydrogel‐based vaccine platform which shows sustained OVA release and enhanced anti‐OVA adaptive immune response relative to bolus control.^1^ A potential advantage of HA cryogel‐based vaccines is that the gels are pre‐formed and injected individually, allowing for more control of dosing and less inter‐operator variability. Our results are also consistent with another approach using nanocellulose hydrogels, which shows sustained release of OVA provides stronger protection against murine lymphoma, than bolus vaccination.^53^ However, nanocellulose hydrogels are surgically implanted, whereas HA cryogel‐based vaccination is administered with a syringe. Similar to other biomaterial‐based vaccines, the HA cryogel‐based vaccine allows for incorporating a range of adjuvants and antigens with the advantage of leveraging an established bioorthogonal crosslinking chemistry that reduces the potential for inactivating vaccine components.^54^, ^55^

The results support the development of a HAC2‐based vaccine for protection against infections or cancers, including in settings of immunodeficiency. However, owing to its xenogeneic origin, OVA can be inherently more immunostimulatory than antigens that might be associated with cancer cells. HAC2‐based vaccine formulations could be designed to utilize tumor cell lysate as a source of tumor antigen, precluding the need to identify a specific tumor antigen and potentially allowing for testing the formulation in multiple tumors types.^56^, ^57^ Alternatively, HAC2‐based subunit vaccines could be developed that contain known tumor antigens such as Trp2 and gp100.^58^, ^59^ HAC2‐based cancer neoantigen vaccines would likely require optimization with multiple TLR agonists and multiple antigen targets to improve vaccine efficacy and breadth of the adaptive immune response. Further, efficacy of HAC2‐based neoantigen vaccines could be assessed in combination with checkpoint inhibitor or chemotherapy.^52^, ^60^, ^61^ Taken together, our results demonstrate the development of a biodegradable, vaccine which may be leveraged to provide greater protection against infections or cancers, including in settings of immunodeficiency.

## 4 METHODS

### 4.1 General methods and statistics

Sample sizes for animal studies were based on prior work without use of additional statistical estimations.^12^ Results were analyzed where indicated using Student *t*‐test, one‐way ANOVA with Dunnett multiple comparison, Kruskal–Wallis test with Dunnett multiple comparison, two‐way ANOVA with Bonferroni test, and log‐rank test using Graphpad Prism software. Alphanumeric coding was used in blinding for pathology samples and cell counting.

### 4.2 Reagents

Sodium hyaluronate was purchased from Acros Organics (MW 1.5–2.2 MDa, lot: A0405554, Supplier 1) and NovaMatrix (MW 1.2–1.9 MDa, Pharma Grade 150, lot: 18011 K, Supplier 2). (2‐morpholinoethanesulfonic acid (MES), sodium chloride (NaCl), sodium hydroxide (NaOH), *N*‐hydroxysuccinimide (NHS), 1‐ethyl‐3‐(3‐dimethylaminopropyl)‐carbodiimide hydrochloride (EDC), sodium periodate (311448) and ammonia borane (AB) complex (682098) were purchased from Sigma‐Aldrich. The (4‐(1,2,4,5‐tetrzain‐3‐yl)phenyl)methanamine (tetrazine amine) was purchased from Kerafast (FCC659, lot: 2014). 1‐bicyclo[2.2.1]hept‐5‐en‐2‐ylmethanamine (norbornene amine) was purchased from Matrix Scientific (# 038023, lot: M15S). Cy5‐tetrazine amine was purchased from Lumiprobe (lot: 9D2FH). A total of 1000 Da molecular weight cutoff (MWCO) mPES membrane was purchased from Spectrum (S02‐E001‐05‐N). GM‐CSF was purchased from PeproTech (AF‐315‐03, lot: 081955). CpG (CpG ODN 1826, 5′‐TCC ATG ACG TTC CTG ACG TT‐3′) was purchased from Integrated DNA Technologies (lots: 480037977, 513470982). CpG:Cy5 (CpG ODN 1826, 5′‐Cy5 TCC ATG ACG TTC CTG ACG TT‐3′) was purchased from Integrated DNA Technologies (lot: 526167013). Vaccine grade OVA was purchased from Invivogen (vac‐pova‐100, lot: 5822‐04‐01). Lipopolysaccharide (LPS) was purchased from Sigma‐Aldrich (L3012‐5MG, lot: 0000091258).

### 4.3 Derivatization of HA

Tetrazine functionalized HA (HA‐Tz) or norbornene functionalized HA (HA‐Nb) were prepared by reacting tetrazine amine or norbornene amine to HA using EDC/NHS carbodiimide chemistry. Sodium hyaluronate was dissolved in a buffer solution (0.75% wt/vol, pH ~ 6.5) of 100 mM MES buffer. NHS and EDC were added to the mixture to activate the carboxylic acid groups on the HA backbone followed by either tetrazine amine or norbornene amine. HA from both suppliers was assumed to be 1.8 MDa for purposes of conjugation reactions. To synthesize HA‐Tz, the molar ratios of HA:EDC:NHS:tetrazine are 1:25,000:25,000:2500. To synthesize HA‐Nb, the molar ratios of HA:EDC:NHS:norbornene are 1:25,000:25,000:2500. Each reaction was stirred at room temperature for 24 h and transferred to a 12,000 Da MW cutoff dialysis sack (Sigma Aldrich) and dialyzed in 4 L of NaCl solutions of decreasing molarity (0.125, 0.100, 0.075, 0.050, 0.025, 0, 0, 0, and 0 M) for 8 h per solution. After dialysis, solutions containing HA‐Tz or HA‐Nb were frozen overnight and lyophilized (Labconco Freezone 4.5) for 48 h. Cy5 conjugated HA‐Nb (Cy5‐HA‐Nb) was synthesized following a previously described technique.^12^ A total of 0.8 mg of Cy5‐Tz was reacted with 100 mg of HA‐Nb at 0.2 wt/vol in DI water for 24 h at 37°C and purified by dialysis in DI water using a 12,000 Da MW cutoff dialysis sack for 48 h. Dialysis water bath was changed every ~8 h. The Cy5‐HA‐Nb solution was then frozen overnight and lyophilized for 48 h.

### 4.4 Cryogel formation

We followed a previously described cryogelation method.^12^ To form cryogels, aqueous solutions of 0.6% wt/vol HA‐Tz and Cy5‐HA‐Nb were prepared by dissolving lyophilized polymers into deionized water and left on a rocker at room temperature for a minimum of 8 h to allow for dissolution. The aqueous solutions were then pre‐cooled to 4°C before crosslinking to slow reaction kinetics. HA‐Tz and HA‐Nb solutions were mixed at a 1:1 volume ratio, pipetted into 30 μL Teflon molds which were pre‐cooled to −20°C, and quickly transferred to a −20°C freezer to allow for overnight cryogelation. To form OVA‐HA cryogels, HA‐Tz and Cy5‐HA‐Nb were solubilized in 3.33 mg/mL OVA solution in DI water prior to crosslinking. To form HA cryogel vaccines, 1 μL/cryogel of CpG (100 mg/mL concentration) and/or GM‐CSF (1 mg/mL concentration) was added to HA‐Tz or Cy5‐HA‐Nb solubilized in OVA solution prior to crosslinking.

### 4.5 Endotoxin testing

Endotoxin testing was conducted using a commercially available endotoxin testing kit (88282, Thermo Fisher Scientific, lot: VH310729) and following manufacturer's instructions. HA‐Tz and Cy5‐HA‐Nb from both suppliers were solubilized at 0.3 wt% in endotoxin free water and samples were tested in technical triplicates. To calculate endotoxin content of a single HA cryogel, the EU/mL concentration for HA‐Tz and Cy5‐HA‐Nb were multiplied by 0.03 (30 μL of volume per HA cryogel). Other components of the HA cryogel vaccine formulations tested for endotoxin included CpG (100 μg/mL concentration), OVA (100 μg/mL concentration), GM‐CSF (1 μg/mL concentration), and RO water which was used to solubilize all vaccine components.

### 4.6 Pore size analysis of HA cryogels

For scanning electron microscopy (SEM), frozen HA cryogels were lyophilized for 24 h in their molds. Lyophilized HA cryogels were adhered onto sample stubs using carbon tape and coated with iridium in a sputter coater. Samples were imaged using secondary electron detection on a FEI Quanta 250 field emission SEM in the Nano3 user facility at UC San Diego. Fluorescence images of Cy5‐labeled HA cryogels were acquired using a Leica SP8 All experiments were performed at the UC San Diego School of Medicine Microscopy Core. Pore size quantification of SEM images was quantified using FIJI image processing package.^62^

### 4.7 LPS doping of HAC2

LPS was added to HA‐Tz and HA‐Nb solutions from supplier 2 at either 12.5 ng/100 mg or 1.25 ng/100 mg. The mixtures were stirred for 24 h and then frozen overnight and lyophilized for 48 h. Lyophilized polymers were rehydrated to assess endotoxin concentration prior to conducting in vivo studies.

### 4.8 HA cryogel pore‐interconnectedness analysis

Cy5‐labeled HA cryogels were incubated in 1 mL of FITC‐labeled 10 μM diameter melamine resin micro particles (Sigma Aldrich) at 0.29 mg/mL concentration on a rocker at room temperature overnight. Fluorescence images of HA cryogels with FITC‐labeled microparticles were acquired using a Leica SP8 confocal. Interconnectedness of the HA cryogels was determined by generating 3D renderings of confocal z‐stacks using FIJI imaging processing package and assessing fluorescence intensity of both the Cy5 and FITC channels with depth starting from the top of the HA cryogel. All experiments were performed at the UC San Diego School of Medicine Microscopy Core.

### 4.9 In vitro degradation of HA cryogels

Cy5‐labeled HA cryogels were placed into individual 1.5 mL microcentrifuge tubes (3448, Thermo Scientific, lot: 20430852) with 1 mL of 100 U/mL Hyaluronidase from sheep testes Type II (HYAL2, H2126, Sigma Aldrich, lot: SLBZ9984) in 1× phosphate buffered saline (PBS). Degradation studies were conducted in tissue culture incubators at 37°C. Supernatant from samples were collected by centrifuging the samples at room temperature at 2000*g* for 5 min and removing 0.9 mL of supernatant. HA cryogels were resuspended by adding 0.9 mL of freshly made 100 U/mL HYAL2 in 1× PBS. Fluorescence measurements were conducted using a Nanodrop 2000 Spectrophotometer (Thermo Fisher Scientific) and these values were normalized to sum of the fluorescence values over the course of the experiment.

### 4.10 In vitro release assays

OVA‐HAC1, OVA‐HAC2, CpG‐OVA‐HAC2, or GMCSF‐OVA‐HAC2 were placed into individual 1.5 mL microcentrifuge tubes in either 1 mL of 1× PBS or 100 U/mL HYAL2 solubilized in 1× PBS. Quantification of in vitro OVA release was conducted using a Micro BCA kit (Thermo Fisher Scientific, lot: UD277184) on supernatant samples. Absorbance values of 100 U/mL HYAL2 solution was assessed and subtracted from absorbance values in supernatant containing HYAL2 solution. Quantification of in vitro CpG release was conducted using OliGreen Assay kit (Thermo Fisher Scientific, lot: 2471822). Quantification of in vitro GM‐CSF release was conducted using Murine GM‐CSF ELISA kit (Peprotech, lot: 1010055).

### 4.11 Cell lines and cell culture

The B16‐OVA cell line was obtained from Professor Liangfang Zhang's laboratory (University of California, San Diego). The cells were cultured in Dulbecco's modified eagle medium (DMEM, Gibco, lot: 2060444) supplemented with 10% fetal bovine serum (Gibco), 1% penicillin–streptomycin (Corning, lot: 30002357), and 300 μg/mL hygromycin B (Corning, lot: 30240136, selective antibiotic for OVA‐expressing cells).

### 4.12 In vivo mouse experiments

All animal work was conducted at the Moores Cancer Center vivarium at UC San Diego and approved by the Institutional Animal Care and Use Committee (IACUC) under protocol number S17160. All animal experiments followed the National Institutes of Health guidelines and relevant AALAC‐approved procedures. Female C57BL/6J mice (B6, Jax no. 000664) and BALB/cJ mice (Jax no. 000651) were 6–8 weeks at the start of the experiments. Male B6 and B6.129S‐Cybbtm1Din (gp91^phox−^, Jax no. 002365) mice were 6–8 weeks old at the start of experiments. All mice in each experiment were age and sex matched and no randomization was performed. This work did not assess if there are sex‐differences associated with the observed effects. Female mice are commonly used in mouse model of melanoma. Previous studies have shown sex‐based differences in adaptive immune response to both vaccination and tumor growth rate.^63^, ^64^ Females typically develop higher antibody titers than males and tumor growth rate in murine melanoma is slowed in female mice due to a more robust adaptive immune response. It is unknown if there are sex‐differences in the mouse HSCT model described in this work. Male mice were used in the gp91^phox−^ studies as the mutation is X‐linked and males but not females lack superoxide production.^25^, ^26^

### 4.13 Subcutaneous cryogel administration

HA cryogels suspended in 200 μL of sterile 1× PBS were administered into the dorsal flank of mice by means of a 16 G needle positioned approximately midway between the hind‐ and forelimbs. The site of injection was shaved and wiped with a sterile alcohol pad prior to gel injection.

### 4.14 In vivo cryogel degradation

In vivo OVA‐HAC1:Cy5 and OVA‐HAC2:Cy5 degradation studies were performed in B6 mice and gp91^phox−^ mice. In all cases, Cy5‐labeled cryogels were administered into the dorsal flank of an anesthetized mouse and the fluorescent intensity of the HA cryogel was quantified using an IVIS spectrometer (PerkinElmer) at predetermined timepoints and analyzed using LivingImage software (PerkinElmer). At each timepoint, mice were anesthetized and the area around the subcutaneous cryogel was shaved to reduce fluorescence signal attenuation. Fluorescence radiant efficiency, the ratio of fluorescence emission to excitation, was measured longitudinally as a metric to quantify fluorescence from subcutaneous cryogels. These values were normalized to the measured signal on day 3. All experiments were performed at the Moores Cancer Microscopy Core Facility at UC San Diego Health using an IVIS spectrometer.

### 4.15 In vivo release

For in vivo release assays, OVA:Cy5 was prepared by reacting OVA with sulfo‐Cy5 NHS ester (Lumiprobe, lot: 7FM7C) at a 1:50:5 molar ratio of OVA:EDC:Sulfo‐Cy5 NHS ester in MES buffer to form OVA:Cy5. Unreacted EDC and sulfo‐Cy5 NHS ester were removed by overnight dialysis using a 12,000 Da MWCO dialysis membrane (D6191, Sigma Aldrich, lot: SLCL5005). A total of 100 μg of OVA:Cy5 was added to either HAC1 or HAC2 polymer mixture prior to crosslinking. To make CpG:Cy5‐OVA‐HAC2, 100 μg of CpG:Cy5 was added to OVA‐HAC2 polymer mixture prior to crosslinking. For all in vivo release assays, release was assessed normalized to a 6‐h initial timepoint in an analogous manner to in the in vivo cryogel degradation studies in the previous section.

### 4.16 Detection of serum anti‐OVA IgG1 antibody titers

Blood was first collected from the tail vein of mice into EDTA coated tubes (365974, BD, lot: 2181885). Blood was centrifuged at 2000*g* for 10 min at room temperature for serum collection. Anti‐OVA IgG1 antibody titers were quantified using ELISA following established protocol.^42^ High‐binding (3590, Corning) ELISA plates were coated with 1 μg/mL OVA in PBS at 4°C overnight. Serum samples were diluted ranging from 1:8 to 1:1:163,840 and incubated with the plates at room temperature for 1.5 h before staining for mouse IgG1 (406604, Biolegend, lot: B270354). The anti‐OVA titer was defied as the lowest serum dilution with an optical density value above 0.2.

### 4.17 Flow cytometry analysis

Anti‐mouse antibodies to CD45 (30‐F11, lot: B280746), CD11b (M1/70, lot: B322056), Ly6‐G/Gr‐1 (1A8, lot: B259670), CD115 (CSF‐1R, lot: B291837), CD11c (N418, lot: B346713), CD4 (RM4‐5, lot: B240051), CD8α (53‐6.7, lot: B266721), and B220 (RA3‐6B2, lot: B298555) were purchased from Biolegend. Anti‐mouse F4/80 (BM8, lot: 2229150) and was purchased from eBioscience. SIINFEKL tetramer (lot: 57396) was sourced from the NIH tetramer core. All cells were gated based on forward and side scatter characteristics to limit debris, including dead cells. Aqua Zombie fixable viability kit (423102, Biolegend, lot: B348291) was used to separate live and dead cells. Antibodies were diluted 1:400 v/v in staining buffer in and added to cells in 1:1 v/v ratio. Cells were gated based on fluorescence‐minus‐one controls, and the frequencies of cells staining positive for each marker were recorded. To quantify infiltrating immune cells within HA cryogels, spleen, and axillary LNs, mice were sacrificed, organ or HA cryogel was removed, and crushed against a 70‐μ filter screen before antibody staining. All flow cytometry experiments were performed using a Attune® NxT Acoustic Focusing cytometer analyzer (A24858) at UC San Diego. Flow cytometry was analyzed using FlowJo (BD) software.

### 4.18 Histology

After euthanasia, HA cryogels were explanted and fixed in 4% paraformaldehyde (PFA) for 24 h. Exactly 4% PFA was prepared by diluting 16% PFA stock (28908, Thermo Fisher Scientific, lot: XB340632) in 1× PBS. The fixed HA cryogels were then transferred to 70% ethanol solution. Samples were routinely processed, and sections (5 μm) were stained and digitized using an Aperio AT2 Automated Digital Whole Slide Scanner by the Tissue Technology Shared Resource at the Moores Cancer Center at UC San Diego Health. Digital slides were rendered in QuPath and positive cell detection was used to quantify the total number of mononuclear cells within each image. Quantification of mononuclear cell density was determined for each histological section. To quantify fibrotic capsule thickness, Qupath was used to measure the epithelial cell layer starting at the edge of the cryogel. Four measurements per cryogel were taken and all measurements were pooled for analysis.

### 4.19 Hematopoietic stem cell transplant

Irradiations were performed with a Cesium‐137 gamma‐radiation source irradiator (J.L. Shepherd & Co.). Syngeneic HSCT (B6 donor and recipient) and allogenic HSCT (BalbC donors, B6 recipients) consisted of two doses of 5000 cGy 6‐h apart +15 M whole bone marrow cells and 10 M splenocytes. Bone marrow cells for transplantation (from donors) were harvested by crushing all limbs with a mortar and pestle, diluted in 1× PBS, filtering the tissue homogenate through a 70 μm mesh and preparing a single‐cell suspension by passing the cells in the flowthrough once through a 20‐gauge needle. Splenocytes were collected by crushing spleens against a 70 μm mesh and preparing a single‐cell suspension by passing the cells in the flowthrough once through a 20‐gauge needle. Total cellularity was determined by counting cells using a hemocytometer. Subsequently, cells were suspended in 100 μL of sterile 1× PBS and administered to anesthetized mice via a single retroorbital injection. All experiments were performed at the Moores Cancer Animal Facility at UC San Diego Health.

### 4.20 Prophylactic immunization

CpG‐OVA‐HAC2 were administered to mice in a prime and boost schedule 11‐days apart. Unvaccinated mice were used as a control. Mice were challenged 21 days post‐prime with 100K B16‐OVA cells in 100 μL cold PBS injected subcutaneously below the neck using an insulin syringe. Tumor growth was monitored using calipers. Tumor volume was calculated assuming an ellipsoid (Volume = 4/3 × π × long axis/2 × short axis/2 × height/2). Mice were euthanized when the tumor volume exceeded 1200 mm^3^.

### 4.21 Therapeutic immunization

A total of 100K B16‐OVA cells in 100 μL cold PBS injected subcutaneously below the neck using an insulin syringe. CpG‐OVA‐HAC2 were administered to mice 3‐days post tumor inoculation in a prime and boost schedule 11‐days apart. Tumor growth was monitored using calipers. Tumor volume was calculated assuming an ellipsoid (Volume = 4/3 × π × long axis/2 × short axis/2 × height/2). Mice were euthanized when the tumor volume exceeded 1200 mm^3^.

## AUTHOR CONTRIBUTIONS

**Matthew D. Kerr:** Conceptualization, methodology, validation, formal analysis, investigation, resources, data curation, writing – original draft, writing – review & editing and visualization. **Wade T. Johnson:** Investigation, writing – review and editing. **David A. McBride:** Investigation and writing – review and editing. **Arun K. Chumber:** Investigation and writing – review and editing. **Nisarg J. Shah:** Conceptualization, writing – original draft, writing – review and editing, supervision, project administration, funding acquisition. All authors reviewed the manuscript and data, provided input and approved the submission.

## Supporting information


**Table S1.** Endotoxin content of HAC2 with added LPS. Measured endotoxin content of HA‐Tz and Cy5‐HA‐Nb from HA supplier 2 with and without added LPS. The expected HA cryogel average endotoxin content was calculated from the measurements.

**Table S2.** Endotoxin content of adjuvanted‐HAC2 vaccine components. Measured endotoxin content of vaccine components used in the formulation of adjuvanted‐HAC2.

**Figure S1.** Extended HA cryogel characterization. (a) Schematic for tetrazine (Tz) and norbornene (Nb) functionalization of HA polymer, Cy5 functionalization of Nb functionalized HA polymer (Cy5‐HA‐Nb) and crosslinking of Tz functionalized HA polymer (HA‐Tz) with Cy5‐HA‐Nb to make crosslinked Cy5‐labeled HA cryogels. (b) Quantification of pore diameter in HA cryogels from supplier 1 (HAC1) and supplier 2 (HAC2). (c) Time to 50% fluorescence intensity for OVA‐HAC1:Cy5 and OVA‐HAC2:Cy5. Data in (b) represents mean ± SD of *n* = 3 cryogels. Data in (c) represents mean SD of *n* = 5 cryogels. Data in (b,c) compared using Student *t*‐test on pooled measurements.
**Figure S2.** Extended innate immune cell infiltration characterization in OVA‐HAC1 and OVA‐HAC2. (a) Representative gating strategy to determine identity of innate immune cells. (b) Percentage of Aqua Zombie^−^ (live) cells within OVA‐HAC1 and OVA‐HAC2. (c–g) Quantification of (c) total CD45^+^CD11b^+^ (myeloid), (d) CD45^+^CD11b^+^Ly6G^+^ (neutrophil), (e) CD45^+^CD11b^+^Ly6G^−^CD115^+^ (monocyte), (f) CD45^+^CD11b^+^Ly6G^−^CD115^−^F4/80^+^ (macrophage), and (g) CD45^+^CD11b^+^Ly6G^−^CD115^−^F4/80^−^CD11c^+^ (dendritic) cells (DCs) in LPS‐doped OVA‐HAC2. (h) Infiltrating immune cell lineages plotted as a percentage of myeloid cells in LPS‐doped OVA‐HAC2. (i) Assessment of anti‐OVA IgG1 antibody titers in serum of mice which received a single OVA‐HAC2, 0.0052 EU (low) LPS‐doped OVA‐HAC2, or 0.0533 EU (high) LPS‐doped OVA‐HAC2 administered in a prime and boost setting 11‐days apart. Data in (b) represents mean ± SD of *n* = 9 cryogels. Data in (c–h) represents mean ± SD of *n* = 5 cryogels. Data in (i) represents mean ± SD of *n* = 5 mice. Data in (b) compared using Student *t*‐test. Data in (c–g) compared with one‐way ANOVA with Dunnett multiple comparison. Data in (i) compared using two‐way ANOVA with Bonferroni multiple comparison test.
**Figure S3.** In vitro release profile of CpG and GM‐CSF. (a) Quantification of in vitro CpG release from CpG and OVA encapsulated HA cryogel from supplier 2 (CpG‐OVA‐HAC2) in both 1× PBS and hyaluronidase 2 (HYAL2) solution. (b) Quantification of in vitro GM‐CSF release from GM‐CSF and OVA encapsulated HAC2 (GMCSF‐OVA‐HAC2) in both 1× PBS and HYAL2 solution. Data in (a,b) represents mean ± SD of *n* = 4 cryogels. Data in (a,b) compared using two‐way ANOVA with Bonferroni multiple comparison test.
**Figure S4.** Extended characterization of innate immune cell infiltration in adjuvanted OVA‐HAC2. (a,b) Percentage of Aqua Zombie^−^ (live) cells within OVA‐encapsulated HA cryogel from supplier 2 (OVA‐HAC2), GM‐CSF and OVA encapsulated HAC2 (GMCSF‐OVA‐HAC2), CpG and OVA encapsulated HAC2 (CpG‐OVA‐HAC2), and GM‐CSF, CpG, and OVA encapsulated HAC2 (GMCSF‐CpG‐OVA‐HAC2) (a) 10‐days and (b) 21‐days after injection. (c–g) Quantification of (c) total CD45^+^CD11b^+^ (myeloid) cells, (d) CD45^+^CD11b^+^Ly6G^+^ (neutrophils), (e) CD45^+^CD11b^+^Ly6G^−^CD115^+^ (monocytes), (f) CD45^+^CD11b^+^Ly6G^−^CD115^−^F4/80^+^ (macrophages), and (g) CD45^+^CD11b^+^Ly6G^−^CD115^−^F4/80^−^CD11c^+^ (dendritic) cells in cryogels removed 21‐days after injection. (h) Infiltrating immune cell lineages plotted as a percentage of myeloid cells in cryogels removed 21‐days after injection. Data in (a–g) represents mean ± SD of *n* = 5 mice. Data in (a–g) were compared using one‐way ANOVA with Dunnett multiple comparison.
**Figure S5.** Extended characterization assessing vaccine efficacy of bolus vaccine formulations. (a) Overview schematic for assessing bolus vaccine formulations. (b) Assessment of anti‐OVA IgG1 antibody titers in serum of mice which received a single bolus OVA, bolus GM‐CSF + OVA, bolus CpG + OVA, or bolus GM‐CSF + CpG + OVA injection administered in a prime and boost setting 11‐days apart. (c,d) Representative gating strategy to determine identity of adaptive immune cells in (c) draining axillary lymph nodes (LNs) and (d) spleen. (e,f) Percentage of CD45^+^B220^−^CD8^+^SIINFEKL^+^ (OVA‐specific cytotoxic T cells) of total CD45^+^B220^−^CD8^+^ cells (cytotoxic T cells) in (e) axillary lymph nodes and (f) spleen. Data in (b,e,f) represents mean ± SD of *n* = 5 mice. Data in (b) were compared using two‐way ANOVA with Bonferroni multiple comparison test. Data in (e,f) were compared using one‐way ANOVA with Dunnett multiple comparison. Figure 5a created using Biorender.
**Figure S6.** Supplementary data for dose escalation studies. Percentage of CD45^+^B220^−^CD8^+^SIINFEKL^+^ cells (OVA‐specific cytotoxic T cells) of total CD45^+^B220^−^CD8^+^ cells (cytotoxic T cells) in spleen. Data in represents mean ± SD of *n* = 5 mice and were compared using one‐way ANOVA with Dunnett multiple comparison.

## Data Availability

The datasets generated during and/or analyzed during the current study are available from the corresponding authors on reasonable request.
