# Supplementary material for: Biodegradable scaffolds for enhancing vaccine delivery
Source: Bioeng Transl Med. 2023 Aug 21;8(6):e10591. doi: 10.1002/btm2.10591 (PMC10658593; doi:10.1002/btm2.10591)
Supplement: Supplementary file 1 — Table S1. Endotoxin content of HAC2 with added LPS. Measured endotoxin content of HA‐Tz and Cy5‐HA‐Nb from HA supplier 2 with and without added LPS. The expected HA cryogel average endotoxin content was calculated from the measurements. [file BTM2-8-e10591-s002.pdf]

Supplementary Table 1

Material	Solution weight %	LPS added (ng) / HA (mg)	Sample 1 (EU/mL)	Sample 2 (EU/mL)	Sample 3 (EU/mL)	Average (EU/mL)	EU/cryogel
HA-Tz	0.3	None	0.0126	0.0146	0.0198	0.0157	0.000672
Cy5-HA-Nb	0.3	None	0.0344	0.0265	0.0263	0.0291	
HA-Tz	0.3	0.0125	0.178	0.176	0.176	0.177	0.00520
Cy5-HA-Nb	0.3	0.0125	0.174	0.170	0.166	0.170	
HA-Tz	0.03	0.125	0.188	0.183	0.184	0.185	0.0533
Cy5-HA-Nb	0.03	0.125	0.171	0.169	0.171	0.170	
