# Supplementary material for: Biodegradable scaffolds for enhancing vaccine delivery
Source: Bioeng Transl Med. 2023 Aug 21;8(6):e10591. doi: 10.1002/btm2.10591 (PMC10658593; doi:10.1002/btm2.10591)
Supplement: Supplementary file 2 — Table S2. Endotoxin content of adjuvanted‐HAC2 vaccine components. Measured endotoxin content of vaccine components used in the formulation of adjuvanted‐HAC2. [file BTM2-8-e10591-s001.pdf]

Supplementary Table 2

Material	Sample 1 (EU/mL)	Sample 2 (EU/mL)	Sample 3 (EU/mL)	Average
RO Water	<0.01	<0.01	<0.01	<0.01
CpG-ODN (100μg/mL)	<0.01	<0.01	<0.01	<0.01
GM-CSF (1μg/mL)	<0.01	<0.01	<0.01	<0.01
OVA (vaccine grade, 100μg/mL)	0.0299	0.0280	0.0305	0.0294
HA-Tz (0.3wt%, HA supplier 2)	0.0126	0.0146	0.0198	0.0157
HA-Nb-Cy5 (0.3wt%, HA supplier 2)	0.0344	0.0265	0.0263	0.0291
