# Supplementary material for: Biodegradable scaffolds for enhancing vaccine delivery
Source: Bioeng Transl Med. 2023 Aug 21;8(6):e10591. doi: 10.1002/btm2.10591 (PMC10658593; doi:10.1002/btm2.10591)

Supplementary Figure 1

s1a

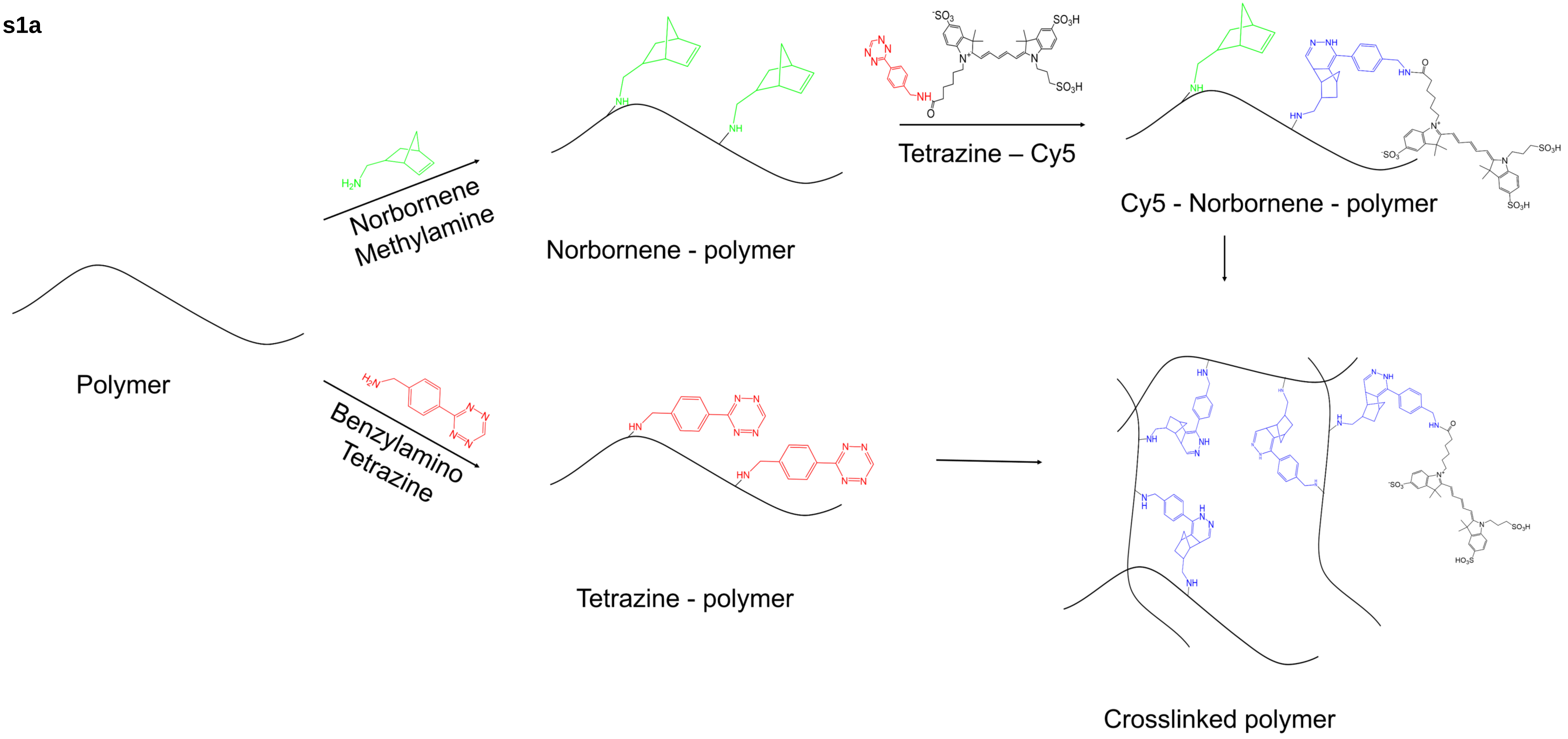

s1b

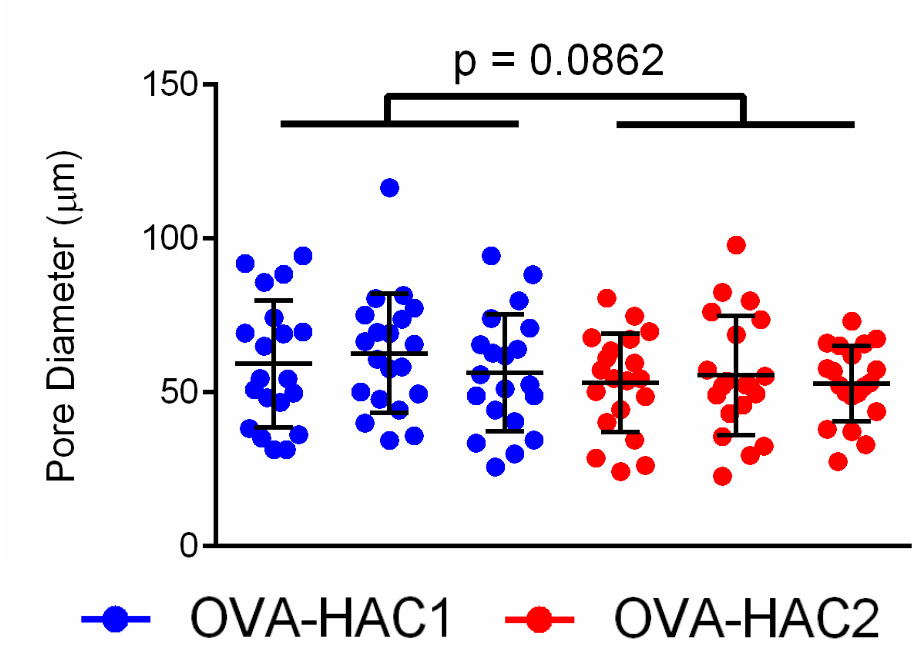

s1c

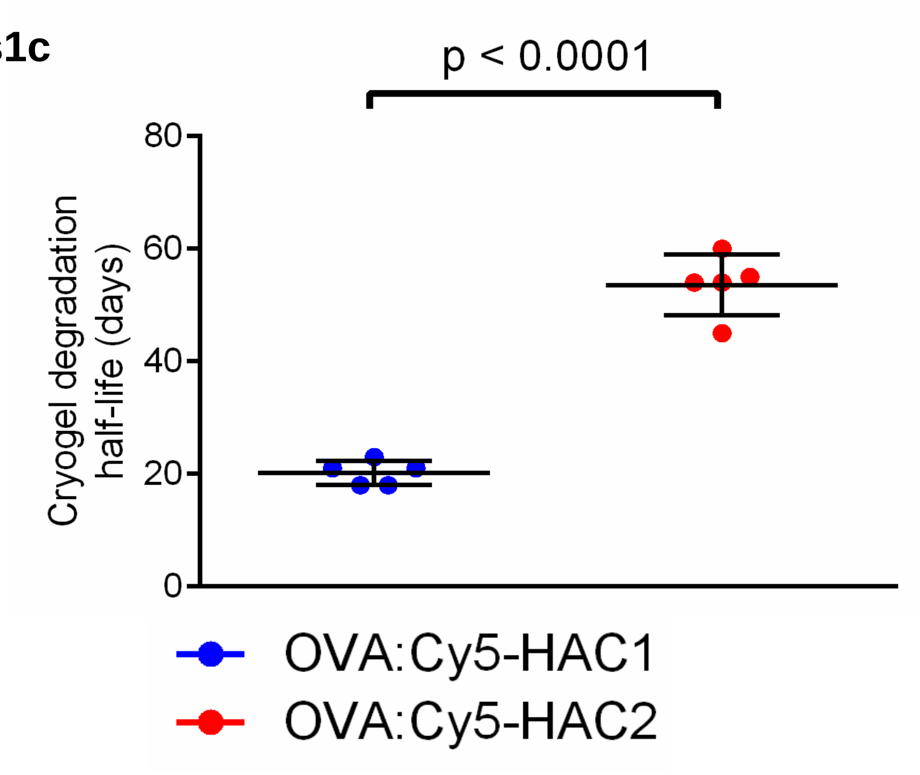

Supplementary Figure 2

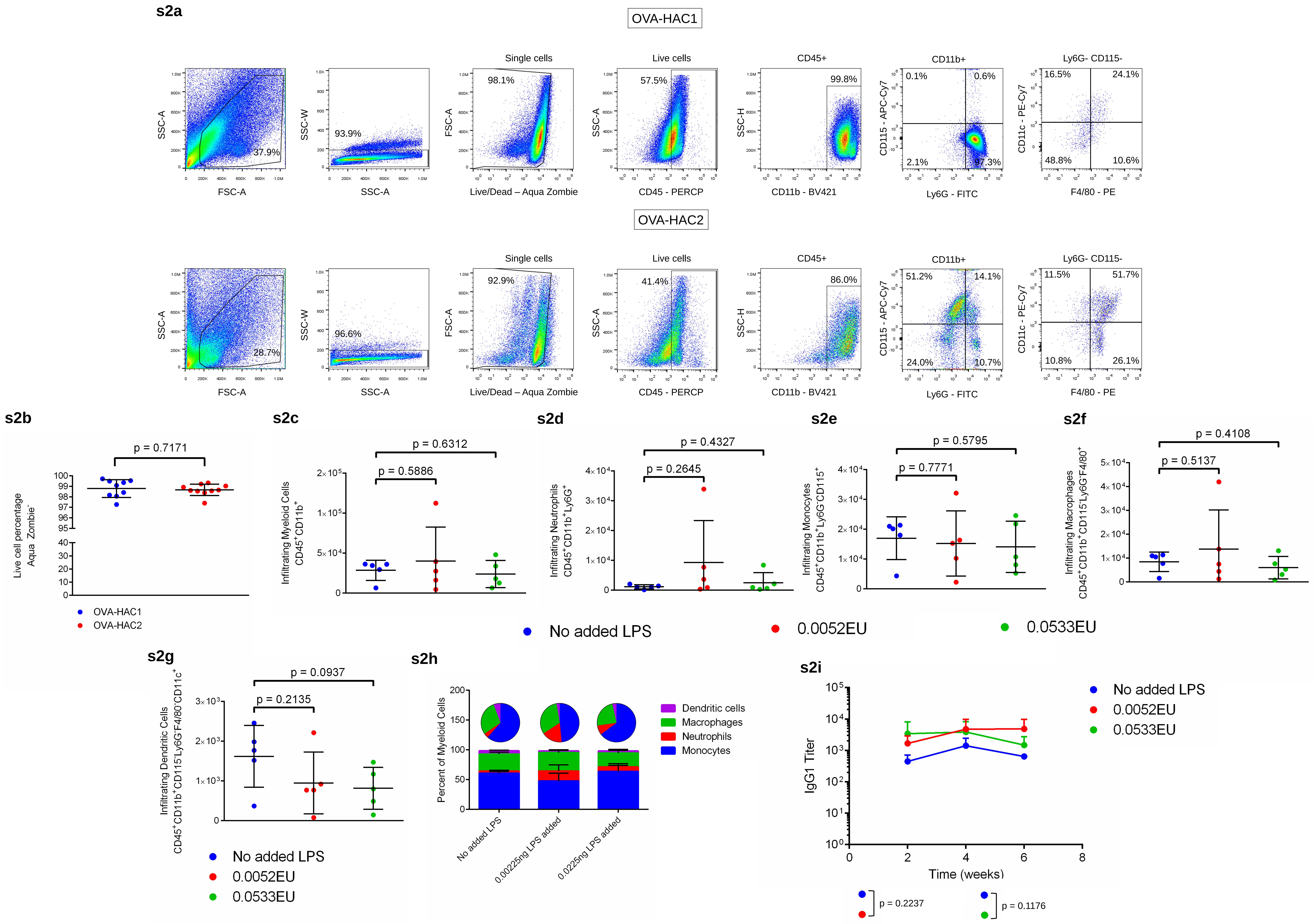

Supplementary Figure 3

s3a **s3b**

In vitro release

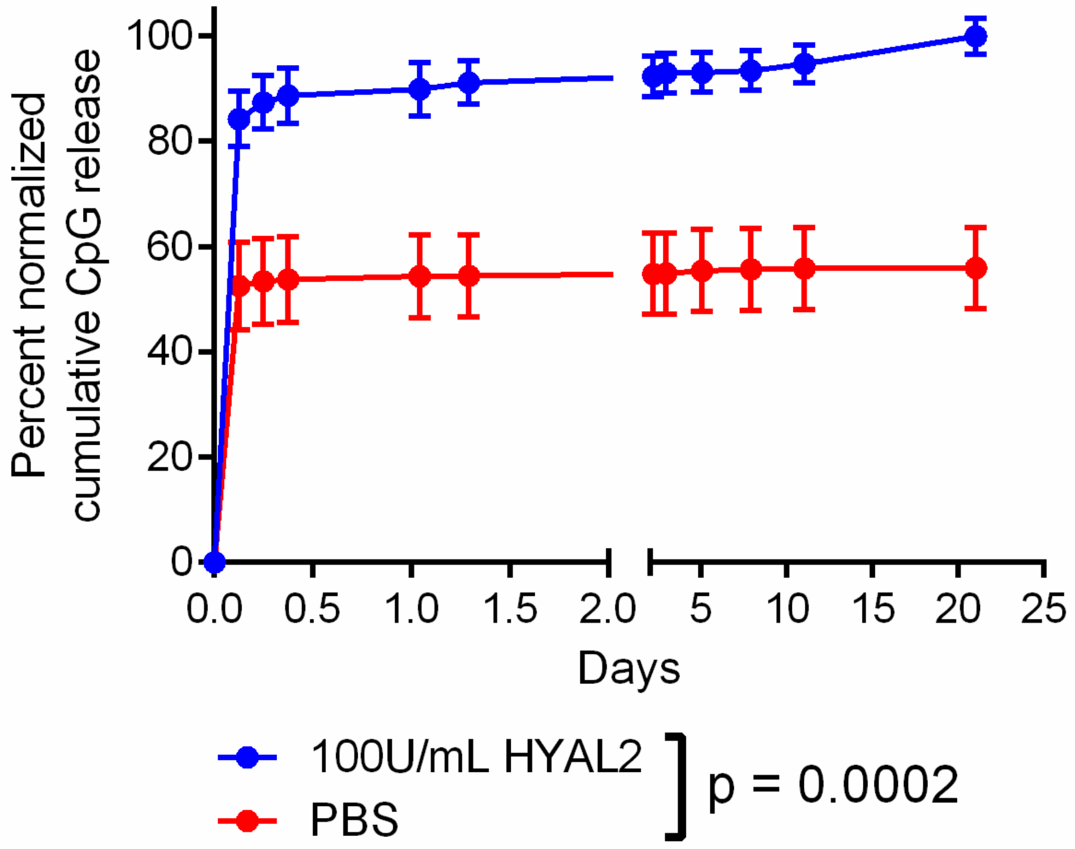

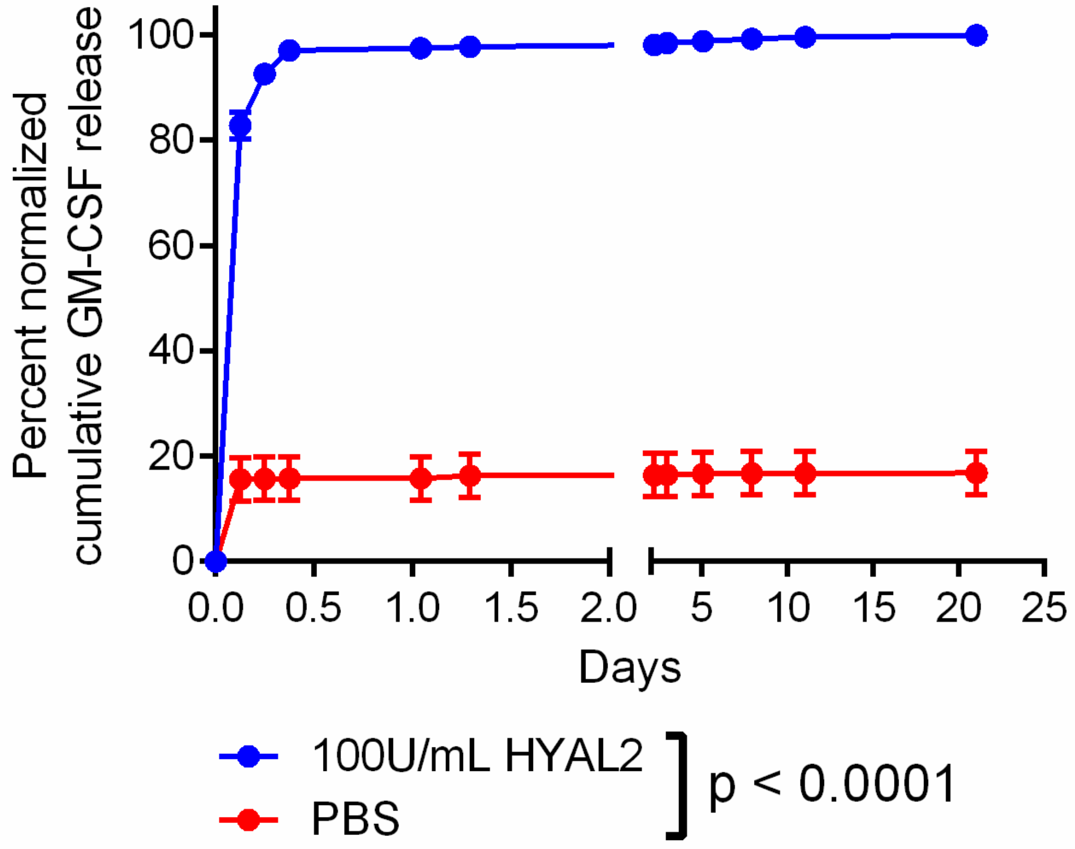

Supplementary Figure 4

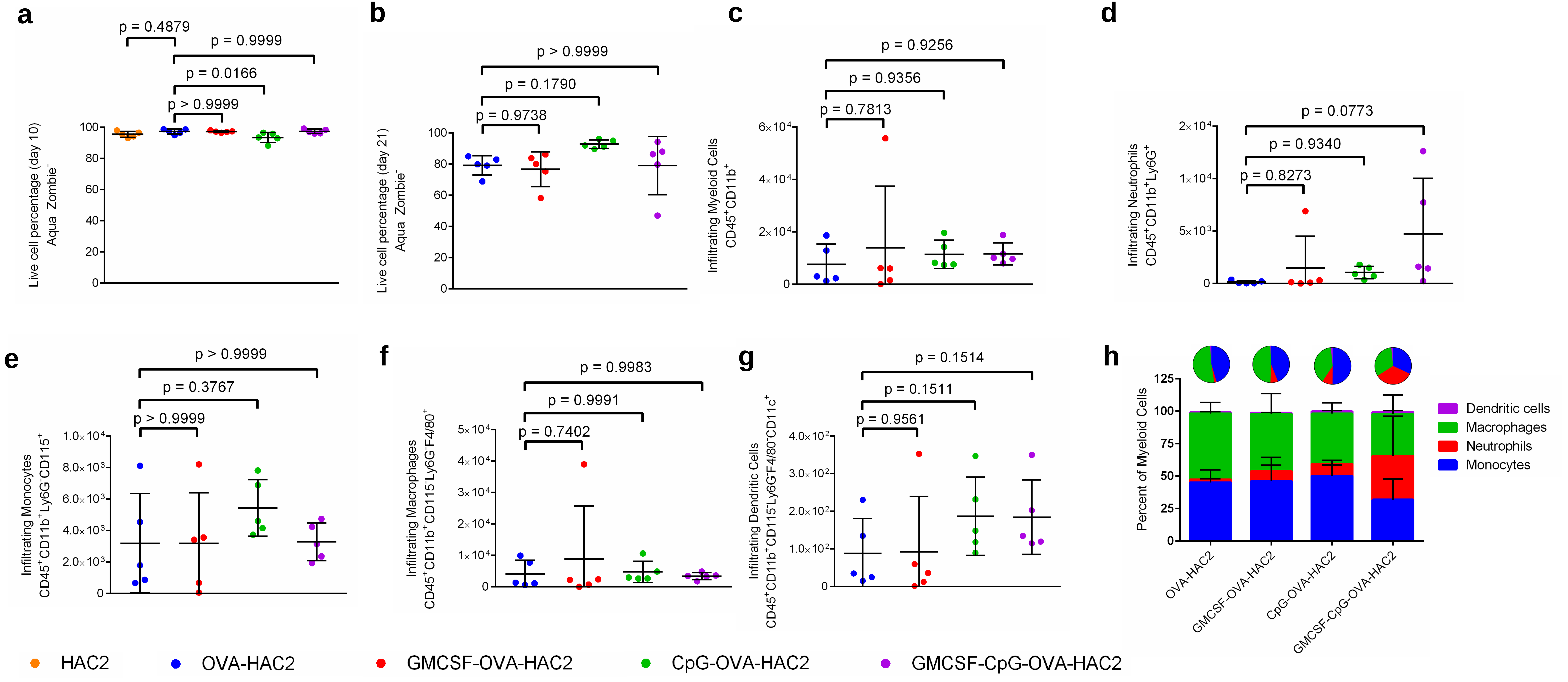

Supplementary Figure 5

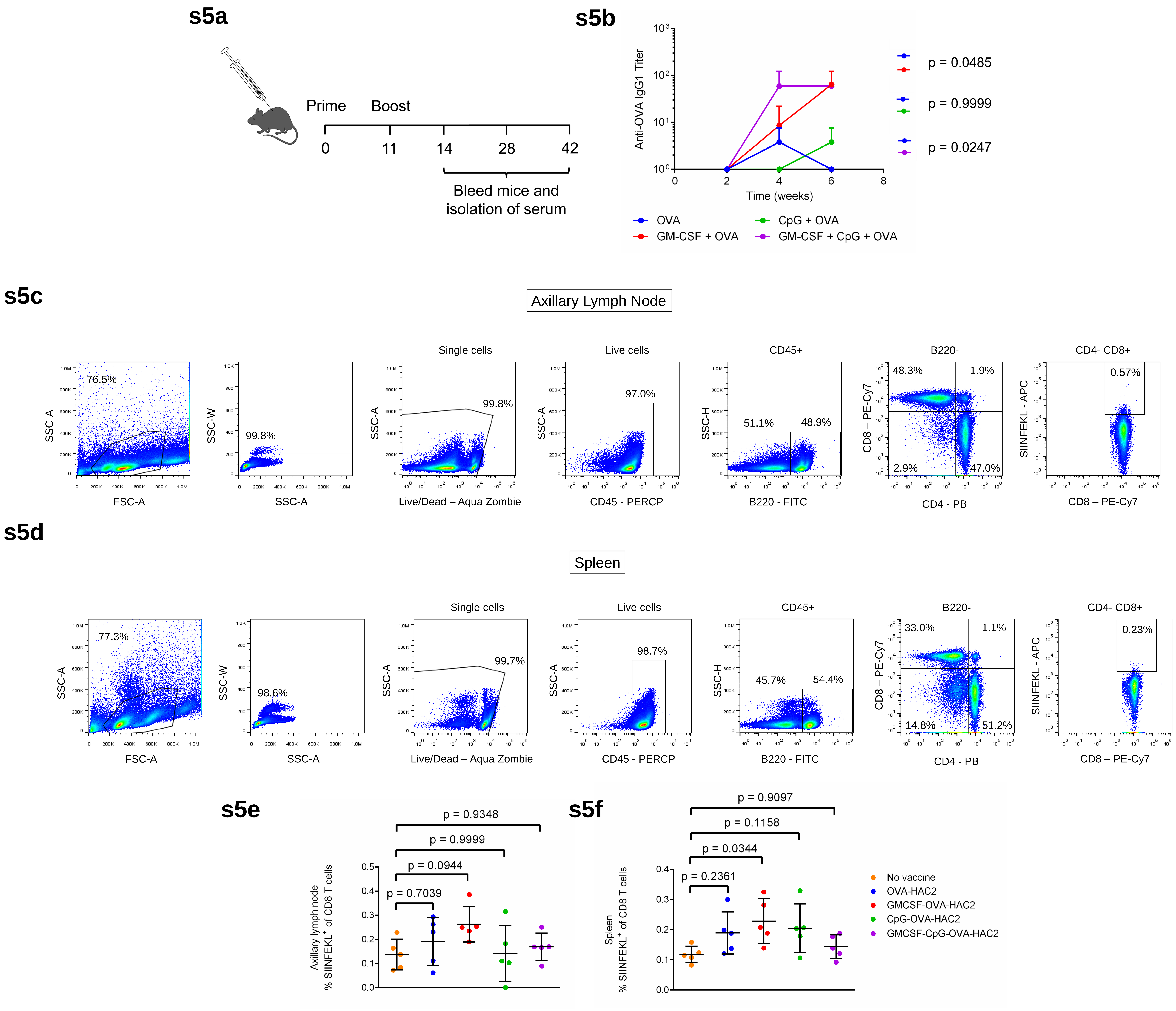

Supplementary Figure 6

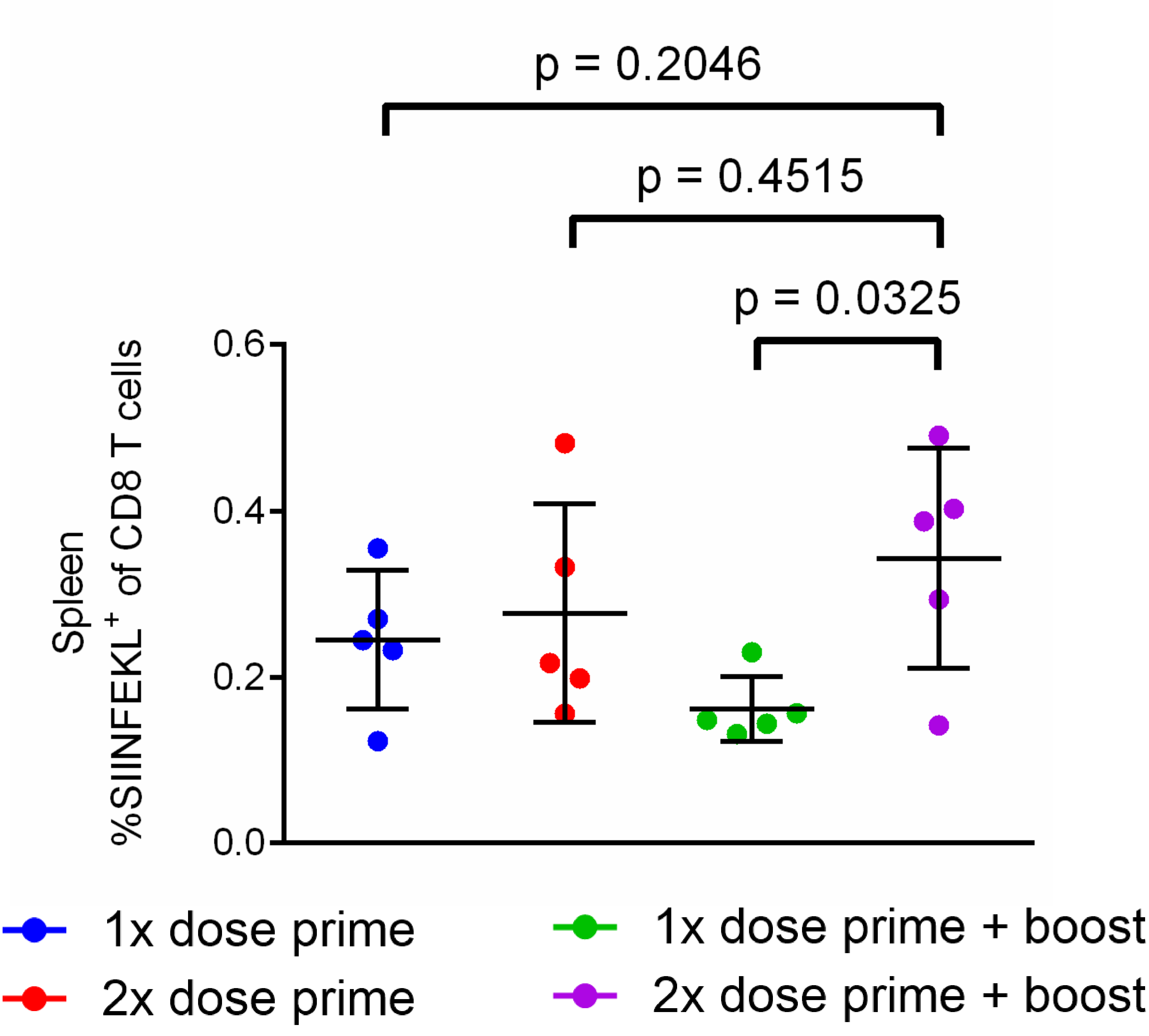

Supplement: Supplementary file 3 — Figure S1. Extended HA cryogel characterization. (a) Schematic for tetrazine (Tz) and norbornene (Nb) functionalization of HA polymer, Cy5 functionalization of Nb functionalized HA polymer (Cy5‐HA‐Nb) and crosslinking of Tz functionalized HA polymer (HA‐Tz) with Cy5‐HA‐Nb to make crosslinked Cy5‐labeled HA cryogels. (b) Quantification of pore diameter in HA cryogels from supplier 1 (HAC1) and supplier 2 (HAC2). (c) Time to 50% fluorescence intensity for OVA‐HAC1:Cy5 and OVA‐HAC2:Cy5. Data in (b) represents mean ± SD of n = 3 cryogels. Data in (c) represents mean SD of n = 5 cryogels. Data in (b,c) compared using Student t‐test on pooled measurements. Figure S2. Extended innate immune cell infiltration characterization in OVA‐HAC1 and OVA‐HAC2. (a) Representative gating strategy to determine identity of innate immune cells. (b) Percentage of Aqua Zombie− (live) cells within OVA‐HAC1 and OVA‐HAC2. (c–g) Quantification of (c) total CD45+CD11b+ (myeloid), (d) CD45+CD11b+Ly6G+ (neutrophil), (e) CD45+CD11b+Ly6G−CD115+ (monocyte), (f) CD45+CD11b+Ly6G−CD115−F4/80+ (macrophage), and (g) CD45+CD11b+Ly6G−CD115−F4/80−CD11c+ (dendritic) cells (DCs) in LPS‐doped OVA‐HAC2. (h) Infiltrating immune cell lineages plotted as a percentage of myeloid cells in LPS‐doped OVA‐HAC2. (i) Assessment of anti‐OVA IgG1 antibody titers in serum of mice which received a single OVA‐HAC2, 0.0052 EU (low) LPS‐doped OVA‐HAC2, or 0.0533 EU (high) LPS‐doped OVA‐HAC2 administered in a prime and boost setting 11‐days apart. Data in (b) represents mean ± SD of n = 9 cryogels. Data in (c–h) represents mean ± SD of n = 5 cryogels. Data in (i) represents mean ± SD of n = 5 mice. Data in (b) compared using Student t‐test. Data in (c–g) compared with one‐way ANOVA with Dunnett multiple comparison. Data in (i) compared using two‐way ANOVA with Bonferroni multiple comparison test. Figure S3. In vitro release profile of CpG and GM‐CSF. (a) Quantification of in vitro CpG release from CpG and OVA encapsulated HA cry [file BTM2-8-e10591-s003.pdf]
